# Long-term survival and the critical role of competing risks in pneumoconiosis: a large-scale retrospective cohort study

**DOI:** 10.3389/fpubh.2026.1782032

**Published:** 2026-03-04

**Authors:** Xinlei Chu, Lang Zhou, Qing Zhou, Lei Chen, Han Liu, Yao Huang, Wenjian Tan, Wei Li, Ning Wang, Lei Han, Ye Li

**Affiliations:** 1 Nanjing Medical University, Nanjing, China; 2 Jiangsu Provincial Center for Disease Control and Prevention (Jiangsu Provincial Academy of Preventive Medicine), Nanjing, China; 3 NHC Key Laboratory of Contraceptives Vigilance and Fertility Surveillance, Jiangsu Health Development Research Center, Jiangsu Provincial Medical Key Laboratory of Fertility Protection and Health Technology Assessment, Nanjing, China; 4 National Institute of Occupational Health and Poison Control, Chinese Center for Disease Control and Prevention, Beijing, China; 5 School of Public Health, Xuzhou Medical University, Xuzhou, China; 6 Key Laboratory of Environmental Medicine Engineering, Ministry of Education, School of Public Health, Southeast University, Nanjing, China; 7 School of Safety Engineering, China University of Mining and Technology, Xuzhou, China

**Keywords:** competing risk, Fine–Gray model, occupational diseases, pneumoconiosis, survival analysis

## Abstract

**Background:**

The extension of survival in patients with pneumoconiosis has led to a shifting mortality spectrum where non-pneumoconiosis causes increasingly act as competing risks. Traditional survival analyses frequently ignore these competing events, potentially biasing prognostic estimates.

**Methods:**

We conducted a retrospective study of 18,064 patients with pneumoconiosis diagnosed between 1960 and 2024 in Jiangsu Province. The Fine–Gray model was used to identify independent predictors of pneumoconiosis-related death while accounting for competing mortality. We compared this evidence with the standard Cox proportional hazards model and established a prognostic nomogram.

**Results:**

The cumulative incidence of non-pneumoconiosis-related death progressively surpassed that of pneumoconiosis-related death during long-term follow-up. Older age at diagnosis, silicosis, an earlier era of diagnosis, and advanced baseline stage were identified as independent risk factors. The traditional Cox model overestimated risk effects for variables with differential impacts on competing outcomes. Subgroup analyses showed a significant interaction between disease type and stage regarding competing mortality risk. Specifically, patients with Stage II silicosis exhibited higher systemic vulnerability compared with those with coal workers’ pneumoconiosis. The constructed nomogram demonstrated high discrimination and calibration.

**Conclusion:**

Non-pneumoconiosis-related death constitutes a critical competing risk that substantially affects the long-term survival outcomes of patients with pneumoconiosis. The Fine–Gray model provides accurate risk stratification by correcting for potential overestimation bias. Clinical management strategies must shift from singular pulmonary care to comprehensive health management that addresses comorbidities to improve overall survival outcomes.

## 1 Introduction

Pneumoconiosis is a systemic disease characterized primarily by diffuse pulmonary fibrosis resulting from long-term occupational dust inhalation and retention (1). It is still a major global occupational health challenge (2, 3). The fibrosis in affected individuals is typically progressive, leading to a sustained decline in respiratory function (4). These generally irreversible pathological changes severely impair work capacity and quality of life (5). As the disease duration extends and the stage advances, the cumulative health burden on patients intensifies (6). Therefore, a thorough comprehension of the long-term prognostic profile of the disease is key to improving patient management.

Strengthened occupational health programs and progress in comprehensive clinical management have extended the overall survival of patients with pneumoconiosis (7). This demographic shift has resulted in a more complex spectrum of mortality risks. During long-term follow-up, patients are exposed to mortality risks not only from pneumoconiosis itself but also from competing causes, including malignancies, cardiovascular diseases, and accidents (8). These non-pneumoconiosis-related deaths preclude the observation of the primary outcome. Such events are particularly prominent in older adult populations and long-term cohorts, constituting statistical competing risks (6).

Prognostic assessment has historically depended on the Kaplan–Meier method or the Cox proportional hazards model. These conventional approaches typically treat deaths from non-primary causes as censored observations (9). This procedure rests on the implicit assumption of independent censoring, which postulates that censored individuals share the same risk as those remaining in the cohort (10). However, in populations with a high burden of competing risks, this assumption is frequently violated (11). Disregarding competing risks inevitably leads to an overestimation of the cumulative incidence of the primary outcome (12). Such statistical bias can distort the identification of prognostic factors and undermine the accuracy of clinical decision-making.

Using a large-scale retrospective cohort from Jiangsu Province, this study comprehensively investigated long-term survival outcomes in patients with pneumoconiosis. We differentiated pneumoconiosis-related deaths from competing causes of death and applied the Fine–Gray proportional subdistribution hazard model for analysis. Independent prognostic factors were identified to construct a nomogram facilitating individual risk stratification. Furthermore, we compared these results with those from the traditional Cox proportional hazards model to clarify methodological differences in prognostic assessment. Ultimately, this study intends to offer a solid methodological basis for the precise evaluation of pneumoconiosis prognosis.

## 2 Methods

### 2.1 Study design and subjects

This retrospective cohort study used data from the Jiangsu Pneumoconiosis Follow-up Online Reporting System, which initially comprised 27,239 registered patients. Stringent exclusion criteria were implemented to confirm data quality and reliability. Individuals were excluded if they were lost to follow-up (*n* = 403), had missing cause of death (*n* = 8,537), or presented with logical irregularities in their records (e.g., years of dust exposure exceeding chronological age or death dates preceding diagnosis dates) (*n* = 235). Consequently, a total of 18,064 patients first diagnosed in Jiangsu Province between 1960 and 2024 were included in the final analytical cohort. The detailed selection process is shown in Supplementary Figure S1. All patients in the final cohort (*n* = 18,064) had complete data for variables used in regression models. Patients with missing covariates were excluded during data cleaning. Therefore, complete case analysis was used without imputation. Characteristics of excluded patients are presented in Supplementary Table S1.

Diagnoses and staging were performed at certified medical institutions in Jiangsu Province authorized to diagnose occupational diseases. Diagnostic criteria complied with the national occupational health standards in effect at the time, specifically the Diagnostic Criteria of Occupational Pneumoconiosis (GBZ 70–2002, GBZ 70–2009, GBZ 70–2015) and the Diagnostic Criteria for Pneumoconiosis on X-ray (GB 5906–1997). The diagnostic procedure included a consensus-based evaluation by an expert panel of at least three qualified radiologists. This evaluation incorporated occupational dust exposure history, clinical symptoms, workplace epidemiological data, and high-kilovolt chest X-rays. Based on the severity of pulmonary fibrosis, cases were classified into Stages I, II, or III. The study was approved by the Ethics Committee of the Jiangsu Provincial Center for Disease Control and Prevention (Approval No. JSJK2022-B002-01).

### 2.2 Variable definitions and outcome ascertainment

Follow-up duration was calculated from the date of initial pneumoconiosis diagnosis to either the date of death or the last follow-up. Follow-up was closed on December 31, 2024. Vital status was ascertained through a combination of active telephone follow-up and linkage with the provincial death registration system. Outcomes were ascertained based on underlying cause-of-death codes recorded in death certificates, classified according to ICD-10. Death information was collected through registry linkage, CDC follow-up, and institutional reporting. Underlying cause of death was determined by trained coders following ICD-10 rules. ICD-10 was used because it remains the standard for China’s medical documentation and death registration during the study period. The follow-up system does not systematically track migration status. Patients who could not be contacted through telephone follow-up and had no death records in the provincial registry were classified as lost to follow-up and excluded from the analysis. The final analytical cohort consisted only of patients with verifiable vital status through either confirmed death records or successful telephone contact.

The primary endpoint was pneumoconiosis-related death, defined as deaths where the underlying cause was coded as J60–J65. Non-pneumoconiosis-related death, comprising mortality from any cause other than these specific codes, was classified as competing death (13). This definition was intentionally conservative to maximize specificity for pneumoconiosis as the underlying cause. Patients who survived to the end of follow-up were censored.

Disease types were classified as silicosis, coal workers’ pneumoconiosis (CWP), welder’s pneumoconiosis (WP), or other pneumoconiosis. The “other pneumoconiosis” category included asbestosis, talc pneumoconiosis, and other dust-related lung diseases not classified as silicosis, CWP, or WP. Regions were classified as southern Jiangsu (Nanjing, Suzhou, Wuxi, Changzhou, Zhenjiang), central Jiangsu (Nantong, Yangzhou, Taizhou), or northern Jiangsu (Xuzhou, Huai’an, Yancheng, Lianyungang, Suqian) based on patients’ residential locations.

### 2.3 Construction of the competing risk regression model

The Fine–Gray proportional subdistribution hazards model was applied to account for the risk of competing death. Univariate analyses were initially performed to evaluate crude associations between baseline characteristics and pneumoconiosis-related mortality. Subsequently, to adjust for potential confounders and identify independent predictors, age at diagnosis, gender, industry category, region, disease type, era of diagnosis, baseline stage, and years of dust exposure were entered into the multivariable model. These variables were selected based on clinical relevance and prior evidence of their associations with pneumoconiosis outcomes. Information on smoking status, comorbidities, and workplace-level exposure controls was not systematically available in the registry data. Effect estimates were expressed as subdistribution hazard ratios (SHR) with 95% confidence intervals (CI). Multicollinearity was assessed using the variance inflation factor (VIF); all VIF values were less than 5, indicating the absence of severe multicollinearity (14). Additionally, the proportional hazards assumption was evaluated using log-minus-log survival curves and scaled Schoenfeld residual plots. Detailed results are provided in Supplementary Figures S2, S3.

### 2.4 Construction and validation of the prognostic nomogram

To develop and validate a tool for individualized prognostic assessment, the entire cohort was randomly partitioned into training and validation sets at a 7:3 ratio. Guided by the multivariable Fine–Gray model results and the principle of parsimony, variables demonstrating independent statistical significance (*p* < 0.05) and substantial prognostic contribution were selected. These variables included age at diagnosis, disease type, era of diagnosis, and baseline stage. A nomogram was subsequently established to predict the 3-, 5-, 10-, and 15-year cumulative incidence of pneumoconiosis-related death. Model performance was evaluated based on discrimination and calibration. Discrimination was quantified using the Concordance Index (C-index), with higher values indicating better predictive accuracy. Calibration was assessed by calibration curves to visually demonstrate the agreement between predicted and observed probabilities. Internal validation was performed using bootstrap resampling with 1,000 repetitions to calculate standard bootstrap confidence intervals for the C-index and ensure model robustness.

### 2.5 Sensitivity analyses and methodological comparison

To verify the robustness of the primary findings among different subpopulations, subgroup analyses were conducted, stratified by age at diagnosis (<50 vs. ≥50 years), era of diagnosis (before 2000 vs. 2000 or later), and disease type (silicosis vs. coal workers’ pneumoconiosis). Within each subgroup, the prognostic impact of baseline stage (Stage II and Stage III vs. Stage I) on pneumoconiosis-related death was evaluated. Interaction tests were performed to assess potential effect modification by these stratifying factors. Furthermore, to demonstrate the necessity of employing a competing risk framework, a traditional Cox proportional hazards model was fitted for methodological comparison. In the Cox model, competing deaths were treated as censored observations, with covariates identical to those in the Fine–Gray model. Discrepancies between hazard ratios (HR) from the Cox model and SHRs from the Fine–Gray model were quantified using relative bias, calculated as (|HR−SHR|/SHR) × 100%.

### 2.6 Statistical analysis

The normality of continuous variables was evaluated using the Shapiro–Wilk or Kolmogorov–Smirnov tests. Normally distributed variables were expressed as mean ± standard deviation (SD) and compared using one-way analysis of variance (ANOVA). Non-normally distributed variables were reported as medians (interquartile range [IQR]) and compared using the Kruskal-Wallis test. Categorical variables were summarized as frequencies (percentages) and analyzed using Pearson’s chi-square test. All statistical analyses were conducted using R software (version 4.3.1). The cmprsk package (version 2.2–11) was used for cumulative incidence estimation and Fine–Gray modeling; the survival package (version 3.5–5) for Cox regression; and the rms (version 6.7–0) and regplot (version 1.1) packages for nomogram construction. All tests were two-sided, with statistical significance defined as *p* < 0.05.

## 3 Results

### 3.1 Characteristics of the study population

As presented in Table 1, the final analysis included 18,064 patients with pneumoconiosis. By the end of follow-up, 15,441 (85.5%) patients remained alive, while 626 (3.5%) had died from pneumoconiosis-related causes and 1,997 (11.1%) from competing causes. The cohort was predominantly male (93.4%), with a median age at diagnosis of 52.0 years (IQR: 45.0–62.0 years). The median age at diagnosis was significantly higher in the pneumoconiosis-related death (59.0 years) and competing death groups (60.0 years) compared with the survival group (51.0 years; *p* < 0.001). Regarding occupational exposure, the mining industry predominated (49.8%), followed by manufacturing (25.6%). Geographically, 60.1% of patients were from southern Jiangsu, 36.2% from northern Jiangsu, and 3.7% from central Jiangsu.

**Table 1:** Baseline characteristics of pneumoconiosis patients by outcome status.

Characteristic	Total (*n* = 18,064)	Alive (*n* = 15,441)	Pneumoconiosis death (*n* = 626)	Competing death (*n* = 1997)	*p* value
Gender, n (%)					0.033
Male	16,875 (93.4)	14,406 (93.3)	578 (92.3)	1891 (94.7)	
Female	1,189 (6.6)	1,035 (6.7)	48 (7.7)	106 (5.3)	
Age at diagnosis, years, median (IQR)	52.0 (45.0–62.0)	51.0 (45.0–60.0)	59.0 (50.0–66.0)	60.0 (51.0–67.0)	<0.001
Industry, *n* (%)					<0.001
Mining	9,000 (49.8)	7,726 (50.0)	382 (61.0)	892 (44.7)	
Manufacturing	4,625 (25.6)	4,134 (26.8)	127 (20.3)	364 (18.2)	
Public/social	3,934 (21.8)	3,134 (20.3)	105 (16.8)	695 (34.8)	
Others	505 (2.8)	447 (2.9)	12 (1.9)	46 (2.3)	
Region, n (%)					<0.001
Southern Jiangsu	10,855 (60.1)	9,617 (62.3)	373 (59.6)	865 (43.3)	
Central Jiangsu	665 (3.7)	583 (3.8)	24 (3.8)	58 (2.9)	
Northern Jiangsu	6,544 (36.2)	5,241 (33.9)	229 (36.6)	1,074 (53.8)	
Dust exposure duration, years, median (IQR)	15.0 (7.0–23.0)	15.0 (7.0–22.0)	18.0 (9.0–26.0)	14.0 (5.0–23.0)	<0.001
Disease type, *n* (%)					<0.001
Silicosis	12,275 (68.0)	10,271 (66.5)	460 (73.5)	1,544 (77.3)	
CWP	3,100 (17.2)	2,742 (17.8)	99 (15.8)	259 (13.0)	
WP	1,013 (5.6)	997 (6.5)	5 (0.8)	11 (0.6)	
Other pneumoconiosis	1,676 (9.3)	1,431 (9.3)	62 (9.9)	183 (9.2)	
Stage at diagnosis, *n* (%)					<0.001
I	15,338 (84.9)	13,355 (86.5)	379 (60.5)	1,604 (80.3)	
II	2,158 (11.9)	1,679 (10.9)	174 (27.8)	305 (15.3)	
III	568 (3.1)	407 (2.6)	73 (11.7)	88 (4.4)	
Era of diagnosis, *n* (%)					<0.001
Before 2000	5,883 (32.6)	4,873 (31.6)	273 (43.6)	737 (36.9)	
2000–2010	6,475 (35.8)	5,427 (35.1)	261 (41.7)	787 (39.4)	
After 2010	5,706 (31.6)	5,141 (33.3)	92 (14.7)	473 (23.7)	
Follow-up time, years, median (IQR)	17.0 (10.0–26.0)	18.0 (11.0–27.0)	14.0 (7.0–22.0)	13.0 (7.0–22.0)	<0.001
Disease progression, *n* (%)					<0.001
No	16,757 (92.8)	14,378 (93.1)	532 (85.0)	1847 (92.5)	
Yes	1,307 (7.2)	1,063 (6.9)	94 (15.0)	150 (7.5)	

IQR, interquartile range; CWP, coal workers’ pneumoconiosis; WP, welder’s pneumoconiosis. Continuous variables are presented as median (IQR) and compared using the Kruskal-Wallis test. Categorical variables are presented as *n* (%) and compared using Pearson’s chi-square test. p values represent overall comparisons across the three outcome groups (alive, pneumoconiosis-related death, and competing death). Region: Southern Jiangsu (Nanjing, Suzhou, Wuxi, Changzhou, Zhenjiang), Central Jiangsu (Nantong, Yangzhou, Taizhou), Northern Jiangsu (Xuzhou, Huai’an, Yancheng, Lianyungang, Suqian).

Patients from northern Jiangsu comprised 53.8% of competing deaths versus 36.6% of survivors (*p* < 0.001). Notably, the proportion of miners was highest in the pneumoconiosis-related death group (61.0%), significantly exceeding that in the survival (50.0%) and competing death groups (44.7%). Additionally, the median duration of dust exposure was longest in the pneumoconiosis-related death group (18.0 years) compared with the survival (15.0 years) and competing death groups (14.0 years) (*p* < 0.001). Clinically, silicosis was the most common disease type (68.0%). While 84.9% of the overall population presented with Stage I disease at baseline, the prevalence of Stage II and Stage III disease was markedly higher in the pneumoconiosis-related death group (27.8 and 11.7%, respectively) than in the survival group (10.9 and 2.6%; *p* < 0.001). Disease progression was most frequent in the pneumoconiosis-related death group (15.0%) compared with the survival (6.9%) and competing death groups (7.5%), with statistically significant differences across the three groups (*p* < 0.001). Significant disparities were also observed in the era of diagnosis and baseline stage among the outcome groups. Median follow-up time varied by era [30.0 years (IQR: 26.0–33.0) for before 2000, 17.0 years (15.0–21.0) for 2000–2010, and 8.0 years (5.0–11.0) for after 2010] and by baseline stage [17.0 years (11.0–27.0) for Stage I, 17.0 years (10.0–23.0) for Stage II, and 11.0 years (6.0–17.0) for Stage III] (Supplementary Table S2).

### 3.2 Analysis of cumulative incidence functions

**Figure 1:**
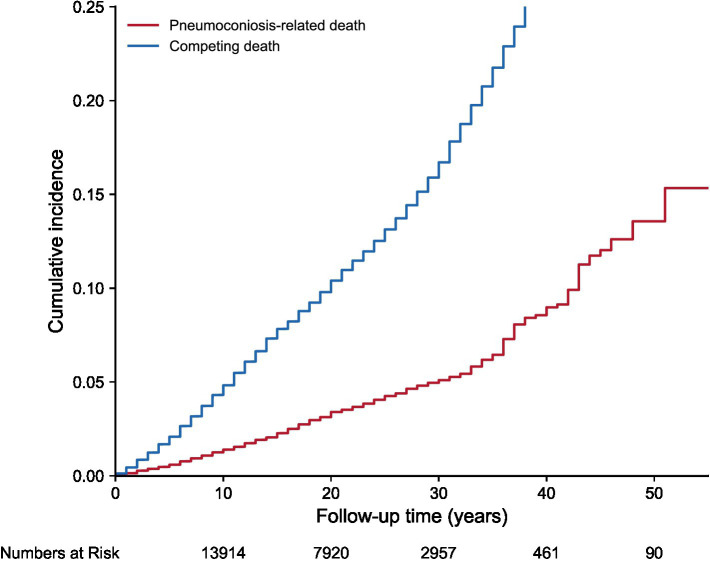
Overall cumulative incidence functions for pneumoconiosis-related death and competing death.

The overall cumulative incidence curves illustrate long-term trends in cause-specific mortality within the study cohort (Figure 1). The analysis indicated that the cumulative incidence of competing death surpassed that of pneumoconiosis-related death throughout the follow-up period. Furthermore, the divergence between the two curves progressively widened over time. Specifically, at 10 years of follow-up, the cumulative incidence of competing death and the event of interest was 4.6 and 1.3%, respectively. By 30 years, these figures rose to 16.3 and 5.0%, respectively. At the 50-year endpoint, the cumulative incidence of competing death reached 39.0%, substantially surpassing the 13.4% observed for pneumoconiosis-related death. These data underscore the substantial burden of competing risks on the prognostic outcomes of this study population.

**Figure 2:**
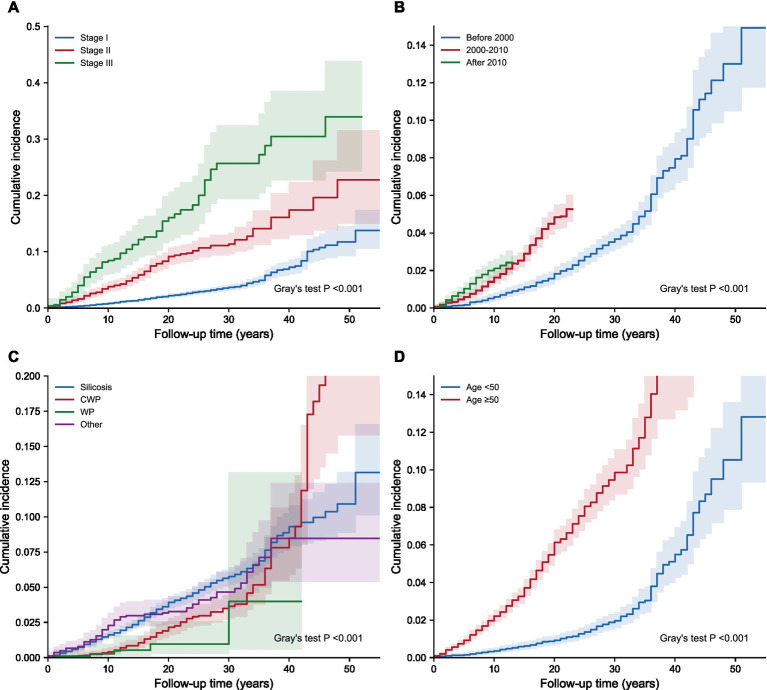
Cumulative incidence functions (CIF) of pneumoconiosis-related death stratified by baseline characteristics. **(A)** CIF by stage at diagnosis. **(B)** CIF by era of diagnosis. **(C)** CIF by disease type. **(D)** CIF by age group at diagnosis.

Stratified analyses of pneumoconiosis-related death revealed significant associations between baseline clinical characteristics and cumulative mortality (Figure 2). In the analysis stratified by baseline stage (Figure 2A), patients with Stage III disease exhibited a steep increase in cumulative mortality, significantly exceeding that of patients with Stage I and Stage II (Gray’s test *p* < 0.001). Distinct temporal disparities were observed for the era of diagnosis (Figure 2B). Compared with patients diagnosed before 2000, those diagnosed after 2010 demonstrated a significantly reduced cumulative incidence of pneumoconiosis-related death (*p* < 0.001). Furthermore, marked prognostic differences existed among disease types (Figure 2C); cumulative mortality for patients with silicosis rose rapidly during early follow-up and was significantly higher than for patients with coal workers’ pneumoconiosis. Age at diagnosis also showed a distinct gradient (Figure 2D), with the older group (≥50 years) showing markedly higher cumulative mortality than the younger group (<50 years) (*p* < 0.001). Supplementary Figure S4 presents the corresponding cumulative incidence curves for competing death stratified by baseline characteristics. Stratified analyses by disease progression and industry categories are provided in Supplementary Figure S5.

Stratified analysis by region revealed contrasting patterns (Supplementary Figure S6). For pneumoconiosis-related death, cumulative incidence curves overlapped across regions (Gray’s test *p* = 0.168). For competing death, northern Jiangsu showed significantly higher cumulative incidence than southern regions (Gray’s test *p* < 0.001), indicating that regional disparities primarily affect non-pneumoconiosis mortality.

### 3.3 Fine–Gray competing risk regression analysis for pneumoconiosis-related death

Evaluation of the proportional hazards assumption using log-minus-log survival curves (Supplementary Figure S2) and scaled Schoenfeld residual tests (Supplementary Figure S3) showed that although the global test was statistically significant (*p* < 0.001), this primarily reflects the large sample size. Individual covariates including era 2000–2010 (*p* = 0.655), welder’s pneumoconiosis (*p* = 0.736), and central Jiangsu (*p* = 0.047) showed no significant time-varying effects. Log-minus-log curves for stage, era, disease type, region, and age group demonstrated generally parallel patterns, supporting the adequacy of the proportional hazards assumption for the core variables.

Univariate Fine–Gray competing risk regression analysis indicated that age at diagnosis, employment in the manufacturing industry, specific disease types (CWP and WP), era of diagnosis, baseline stage, and years of dust exposure were all significantly associated with the risk of pneumoconiosis-related death (Table 2). In contrast, gender and other industry categories did not show notable correlations at the univariate level.

**Table 2:** Fine–Gray competing risk regression analysis for pneumoconiosis-related death.

Variable	Unadjusted SHR (95% CI)	*P* value	Adjusted SHR (95% CI)	*P* value
Age at diagnosis, years	1.08 (1.07–1.08)	<0.001	1.09 (1.08–1.10)	<0.001
Gender				
Male	Reference		Reference	
Female	1.12 (0.83–1.50)	0.460	0.96 (0.69–1.32)	0.780
Industry				
Mining	Reference		Reference	
Manufacturing	0.81 (0.67–0.99)	0.039	0.92 (0.71–1.19)	0.530
Public/Social	0.97 (0.78–1.20)	0.770	0.66 (0.47–0.92)	0.014
Others	0.73 (0.42–1.28)	0.280	0.86 (0.48–1.56)	0.630
Region				
Southern Jiangsu	Reference		Reference	
Central Jiangsu	1.15 (0.77–1.74)	0.490	1.92 (1.24–2.98)	0.004
Northern Jiangsu	1.15 (0.97–1.35)	0.100	1.44 (1.16–1.78)	<0.001
Disease type				
Silicosis	Reference		Reference	
CWP	0.74 (0.60–0.91)	0.005	0.62 (0.49–0.79)	<0.001
WP	0.29 (0.12–0.69)	0.005	0.82 (0.33–2.04)	0.680
Other pneumoconiosis	1.01 (0.78–1.32)	0.930	1.00 (0.72–1.40)	0.980
Era of diagnosis				
Before 2000	Reference		Reference	
2000–2010	1.56 (1.33–1.83)	<0.001	0.66 (0.54–0.80)	<0.001
After 2010	1.28 (1.03–1.59)	0.027	0.38 (0.28–0.52)	<0.001
Stage at diagnosis				
I	Reference		Reference	
II	2.98 (2.50–3.55)	<0.001	2.94 (2.41–3.60)	<0.001
III	5.47 (4.26–7.02)	<0.001	5.18 (3.91–6.87)	<0.001
Dust exposure duration, years	1.02 (1.01–1.03)	<0.001	1.01 (1.00–1.02)	0.160

SHR, subdistribution hazard ratio; CI, confidence interval; CWP, coal workers’ pneumoconiosis; WP, welder’s pneumoconiosis. SHRs and 95% CIs were estimated using the Fine–Gray proportional subdistribution hazards model. Pneumoconiosis-related death was the event of interest, with non-pneumoconiosis-related death treated as the competing event. Region: Southern Jiangsu (Nanjing, Suzhou, Wuxi, Changzhou, Zhenjiang), Central Jiangsu (Nantong, Yangzhou, Taizhou), Northern Jiangsu (Xuzhou, Huai’an, Yancheng, Lianyungang, Suqian).

In the multivariable model adjusting for all candidate variables, age at diagnosis, regional economic level, disease type, era of diagnosis, and baseline stage emerged as independent predictors of pneumoconiosis-related death. Baseline stage exhibited the strongest prognostic impact, showing a distinct gradient. Compared with Stage I, the adjusted SHRs for pneumoconiosis-related death were 2.94 (95% CI 2.41–3.60) for Stage II and 5.18 (95% CI 3.91–6.87) for Stage III. Age at diagnosis was positively associated with mortality risk; each 1-year increase corresponded to a 9% increase in risk (SHR 1.09, 95% CI 1.08–1.10). Region demonstrated a significant independent effect. Compared with southern Jiangsu, patients from central Jiangsu (SHR 1.92, 95% CI 1.24–2.98) and northern Jiangsu (SHR 1.44, 95% CI 1.16–1.78) exhibited significantly elevated pneumoconiosis-related mortality risk. Marked variations in mortality risk were also identified across disease types. Relative to silicosis, patients with CWP had a 38% lower risk (SHR 0.62, 95% CI 0.49–0.79).

Notably, after multivariable adjustment, the association with the era of diagnosis reversed from a positive to a negative direction compared with the univariate analysis, revealing a significant independent protective effect. Compared with patients diagnosed before 2000, the risk was 34% lower for those diagnosed between 2000 and 2010 (SHR 0.66, 95% CI 0.54–0.80) and 62% lower for those diagnosed after 2010 (SHR 0.38, 95% CI 0.28–0.52). Additionally, employment in public administration and social organizations was associated with lower mortality risk compared with mining (SHR 0.66, 95% CI 0.47–0.92). In contrast, gender, employment in manufacturing or other industries, WP, other pneumoconiosis types, and years of dust exposure did not retain statistical significance in the multivariable model after adjusting for other covariates.

### 3.4 Fine–Gray competing risk regression analysis for competing death

Univariate Fine–Gray regression analysis indicated that age at diagnosis, gender, industry category, region, disease type, era of diagnosis, baseline stage, and years of dust exposure were all significantly associated with the risk of competing death (Supplementary Table S3). In the multivariable model, age at diagnosis, gender, region, disease type, baseline stage, and years of dust exposure emerged as independent predictors. Age at diagnosis was positively associated with mortality risk; each 1-year increase corresponded to a 9% rise in the risk of competing death (SHR 1.09, 95% CI 1.09–1.10). Females exhibited a lower risk than males (SHR 0.77, 95% CI 0.63–0.94). Region demonstrated a strong independent effect on competing mortality. Compared with southern Jiangsu, patients from central Jiangsu (SHR 1.44, 95% CI 1.09–1.91) and northern Jiangsu (SHR 1.90, 95% CI 1.69–2.14) exhibited significantly elevated competing death risk, with northern regions showing a 90% higher risk.

Regarding disease type, relative to silicosis, patients with CWP demonstrated a 46% lower risk (SHR 0.54, 95% CI 0.46–0.64), and those with WP showed a 51% lower risk (SHR 0.49, 95% CI 0.27–0.90). Baseline stage exhibited a significant but modest effect on competing death. Compared with Stage I, both Stage II (SHR 1.49, 95% CI 1.29–1.72) and Stage III (SHR 1.41, 95% CI 1.10–1.80) were associated with increased competing risk. Years of dust exposure showed a weak but statistically significant negative correlation with the risk (SHR 0.99, 95% CI 0.98–1.00). Notably, although industry category and era of diagnosis showed strong associations in the univariate analysis, these associations attenuated and failed to maintain statistical significance in the multivariable model after adjusting for covariates such as age, region, and disease type.

### 3.5 Subgroup analyses and consistency of effects

**Figure 3:**
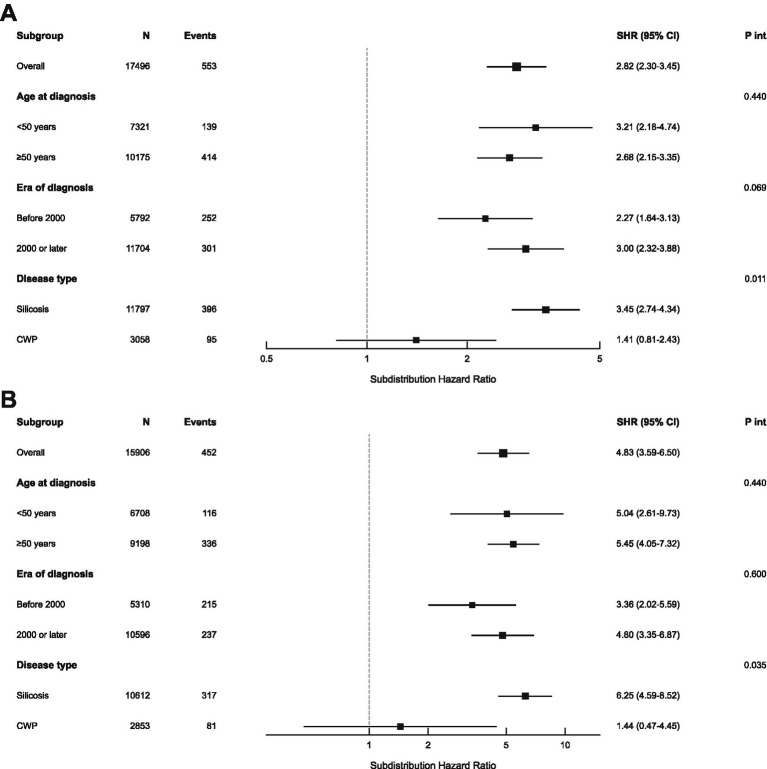
Forest plots of subgroup analysis for pneumoconiosis-related death. **(A)** Adjusted SHRs for Stage II versus Stage I. **(B)** Adjusted SHRs for Stage III versus Stage I.

Subgroup analyses demonstrated that the prognostic impact of baseline stage on pneumoconiosis-related death remained robust across most stratifications (Figure 3). Regarding Stage II (overall SHR 2.82) and Stage III (overall SHR 4.83), no notable interactions were observed with age at diagnosis or era of diagnosis (*P* for interaction > 0.05). However, disease type significantly modified the effect of stage (*P* for interaction = 0.011 for Stage II; *p* = 0.035 for Stage III). Among patients with silicosis, Stage II (SHR 3.45, 95% CI 2.74–4.34) and Stage III (SHR 6.25, 95% CI 4.59–8.52) were associated with substantially elevated mortality risks. In contrast, among patients with CWP, the associations of Stage II (SHR 1.41, 95% CI 0.81–2.43) and Stage III (SHR 1.44, 95% CI 0.47–4.45) with mortality were not statistically significant.

Subgroup analyses regarding competing death showed distinct patterns (Supplementary Figure S7), indicating that the prognostic impact of baseline stage on competing death was generally modest (SHR 1.25–1.37). Notably, significant effect modification by disease type was observed for the comparison between Stage II and Stage I (*P* for interaction = 0.015). Specifically, the risk of competing death was significantly elevated among patients with Stage II silicosis (SHR 1.40, 95% CI 1.20–1.64), whereas no significant association was found for those with CWP (SHR 0.82, 95% CI 0.53–1.25). Furthermore, no significant interaction by disease type was detected for the comparison between Stage III and Stage I.

### 3.6 Construction and validation of the prognostic nomogram for pneumoconiosis-related death

**Figure 4:**
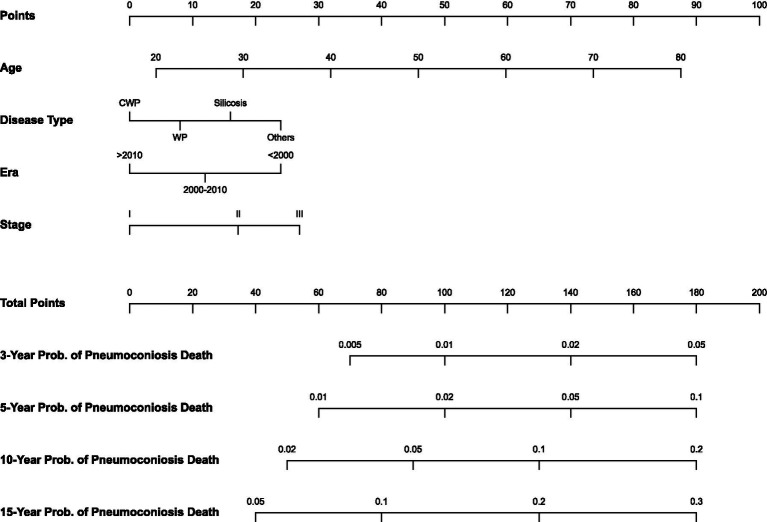
Nomogram for predicting the 3-, 5-, 10-, and 15-year cumulative incidence of pneumoconiosis-related death.

The study population was randomly partitioned into a training cohort (*N* = 12,645) and a validation cohort (*N* = 5,419) at a 7:3 ratio. Guided by the multivariable Fine–Gray model, variables exhibiting considerable prognostic contribution, including age at diagnosis, disease type, era of diagnosis, and baseline stage, were selected to construct a nomogram predicting the 3-, 5-, 10-, and 15-year cumulative incidence of pneumoconiosis-related death (Figure 4). In the nomogram, age at diagnosis and baseline stage spanned the widest point ranges, indicating that these two variables contributed most markedly to individualized prognostic assessment. Validation analysis showed favorable discrimination in both cohorts. The C-indices were 0.769 (95% CI 0.742–0.797) in the training cohort and 0.753 (95% CI 0.714–0.791) in the validation cohort. The consistency between these values suggested the absence of significant overfitting. Calibration analysis showed favorable agreement between predicted and observed probabilities. At the 3-, 5-, 10-, and 15-year time points, the intercepts of the calibration curves were close to 0 (range −0.001 to −0.010) while slopes ranged from 1.10 to 1.33 (Supplementary Figure S8). For calibration assessment, patients in the training cohort were divided into five groups based on quintiles of predicted risk, with each quintile containing approximately 1,069 to 1,099 patients. The wider confidence intervals observed at higher predicted probabilities reflect the lower absolute number of events in these groups. The calibration curves generally aligned with the 45-degree ideal reference line, indicating consistency between model-predicted probabilities and actual results.

### 3.7 Comparative validation of the competing risk model and cox proportional hazards model

Supplementary Table S3 compares parameter estimates between the Fine–Gray and Cox models. The magnitude and direction of discrepancy varied across covariates. The Cox model showed substantial differences for era of diagnosis (relative bias +36.0% for 2000–2010 and +65.6% for after 2010) and moderate differences for baseline stage (+12.5% for Stage II and +5.2% for Stage III). However, several covariates showed minimal discrepancy: age (+3.5%), dust exposure (−0.4%), and regional economic level (+1.9% for central Jiangsu and −2.3% for northern Jiangsu). Some covariates showed negative bias, with Cox producing smaller estimates than Fine–Gray for CWP (−15.8%) and female gender (−9.6%).

## 4 Discussion

Based on a large-scale retrospective cohort, this study comprehensively evaluated the long-term survival outcomes of patients with pneumoconiosis. We confirmed that the competing risk effect of non-pneumoconiosis-related death is non-negligible during long-term follow-up. Using the Fine–Gray model, we identified age at diagnosis, disease type, era of diagnosis, and baseline stage as independent prognostic factors for pneumoconiosis-related death. Compared with the traditional Cox model, the Fine–Gray model yielded more conservative and accurate risk estimates. This finding underscores the methodological importance of employing competing risk models to address survival analysis bias in the context of population aging and a shifting spectrum of causes of death. Notably, while the divergence between competing and pneumoconiosis-related mortality was pronounced at 50 years, these late estimates are based on a smaller number of patients at risk and may be influenced by selective survival of healthier individuals.

Methodologically, this study demonstrates the importance of appropriate model selection for survival analysis in the presence of competing risks. Previous studies have predominantly employed the Cox model while treating non-pneumoconiosis-related deaths as censored data. This approach relies on the implicit assumption that censored individuals share the same risk of pneumoconiosis-related death as those remaining in the cohort. However, in our cohort, the cumulative incidence of non-pneumoconiosis-related death increased significantly with the extension of follow-up time and ultimately surpassed that of pneumoconiosis-related death. The Cox and Fine–Gray models address different estimands. Cox estimates cause-specific hazard ratios, while Fine–Gray estimates subdistribution hazard ratios directly related to cumulative incidence. The magnitude of discrepancy between the two models varied across covariates, with larger differences observed for era of diagnosis and smaller differences for other variables. For occupational disease cohorts characterized by older adult patients and complex comorbidities, the Fine–Gray model provides more appropriate estimates for baseline risk stratification and cumulative incidence prediction.

The era of diagnosis emerged as the strongest independent protective factor in this study. Patients diagnosed after 2010 exhibited a significantly reduced risk of pneumoconiosis-related death. This pattern might be attributed to the optimization of occupational health policies and improvements in clinical management in recent years (15, 16). Following the implementation of the National Occupational Disease Prevention and Control Plan and the expansion of coverage for workers’ compensation insurance, patients have gained access to more timely medical intervention and livelihood support post-diagnosis (17, 18). These improvements in social support systems may delay disease progression and reduce the fatality rate of complications. Additionally, the widespread use of radiographic screening technologies has enabled more patients to be diagnosed at an early stage (19). While this may introduce lead-time bias from a statistical perspective, it objectively secures a window of opportunity for early intervention. However, the strong protective effect for diagnoses after 2010 should be interpreted cautiously given the substantially shorter follow-up time for this cohort (median 8.0 years) compared to earlier eras (median 30.0 years for before 2000). Cumulative incidence estimates for recent diagnoses remain immature, and continued follow-up is needed to confirm whether this mortality reduction persists over longer time horizons.

Regarding disease type, the analysis indicated that the prognosis of patients with silicosis was significantly inferior to that of patients with coal workers’ pneumoconiosis. This difference is associated with the high fibrogenic toxicity of free silica dust (20). Free silica particles can continuously induce the apoptosis of alveolar macrophages and the release of inflammatory cytokines, therefore causing progressive pulmonary fibrosis and respiratory failure (21, 22). In contrast, the cytotoxicity of coal dust is relatively lower (20). Notably, we observed a significant interaction between disease type and stage in the subgroup analysis of non-pneumoconiosis-related death. The risk of death from non-pneumoconiosis causes was significantly increased in patients with Stage II silicosis, whereas this phenomenon was not observed in those with coal workers’ pneumoconiosis. This suggests that silicosis, acting as a systemic disease, may induce systemic chronic inflammation that aggravates the vulnerability of extra-pulmonary organs such as the cardiovascular system (23, 24). This finding implies that clinicians managing patients with silicosis should not only focus on pulmonary lesions but also reinforce surveillance for comorbidities, including cardiovascular diseases.

The nomogram established in this study demonstrated favorable predictive performance. This tool incorporates readily accessible clinical variables, including age at diagnosis, stage, and disease type. By quantifying mortality probabilities at various time points, this model facilitates clinicians’ identification of high-risk populations and the formulation of stratified follow-up strategies. For patients identified as having a higher predicted risk, medical institutions are advised to shorten follow-up intervals and actively monitor for potential complications. This individualized risk assessment tool successfully addresses the current gap in occupational disease clinical practice by providing a quantitative prognostic scoring system. Based on predicted 10-year cumulative incidence, we suggest stratifying patients into low risk (<3%), moderate risk (3–7%), and high risk (>7%) for annual, semi-annual, and quarterly follow-up, respectively.

A notable finding was the significant impact of regional economic level on both mortality outcomes. Patients from northern Jiangsu exhibited 44% higher pneumoconiosis-related death risk and 90% higher competing risk compared with southern regions. These disparities likely reflect differential access to specialized occupational medicine services and general healthcare infrastructure. The minimal bias between Cox and Fine–Gray estimates for region (<3%) versus substantial overestimation for era (+65.6%) underscores that variables with proportional effects across outcomes show similar estimates in both models, whereas those with differential impacts require competing risk methods.

The generalizability of our findings to other regions warrants consideration. While Jiangsu Province features well-developed occupational health infrastructure, the diagnostic criteria and staging systems are standardized nationwide, ensuring consistent disease classification across China. The temporal mortality reduction observed in our cohort aligns with trends reported from other major industrial provinces (16), suggesting that recent policy reforms have yielded broad benefits. However, regional variations in healthcare access and economic development may influence the magnitude of observed effects. Our findings carry important policy implications for China’s occupational health system. The predominance of non-pneumoconiosis-related death highlights the urgent need to shift from disease-specific monitoring to comprehensive health management that addresses cardiovascular and other systemic comorbidities. The prognostic nomogram developed here provides a practical tool for individualized risk stratification and could be integrated into national surveillance platforms to guide resource allocation and personalized follow-up strategies. Furthermore, the regional disparities identified suggest that future policy efforts should prioritize healthcare infrastructure development in economically disadvantaged areas to ensure equitable access to both specialized occupational medicine and general health services.

This study also has certain limitations. First, the exclusion of 9,175 patients (33.7% of the initial registry) introduces potential selection bias. The majority of exclusions were due to missing cause of death, which occurred predominantly in patients who died before implementation of the online reporting system in 2010 or whose deaths occurred outside Jiangsu Province without registry linkage. Excluded patients differed from the analytical cohort in several important dimensions. They were disproportionately diagnosed in earlier eras and had markers of more severe disease, including higher proportions of advanced-stage disease at baseline and disease progression during follow-up. Consequently, our study likely underestimates absolute mortality rates compared to the full registry population. However, the enrichment of severe cases among excluded patients would be expected to increase pneumoconiosis-related mortality in that subgroup, implying that our finding of competing death dominance in long-term survival is conservative rather than overestimated. Our stratified analyses by era demonstrate consistent patterns of increasing competing risk across all diagnostic periods, supporting the robustness of this core finding. Second, our strict J60–J65 definition may classify deaths where pneumoconiosis was a contributing factor (e.g., coded as respiratory failure or cor pulmonale) as competing deaths. This may underestimate pneumoconiosis-related mortality and overestimate competing mortality, making our findings conservative. Finally, several important variables were unavailable. Enterprise-level characteristics (scale, safety practices) and quantitative dust exposure measurements were not systematically documented, limiting exposure-response analyses. While we included years of dust exposure, industry categories, and region as proxies, individual-level socioeconomic indicators (education, income) would provide more nuanced insights. Additionally, detailed smoking history and comorbidity data were unavailable, which may result in residual confounding. The higher competing death risk observed in older patients and public/social sector workers may partly reflect unmeasured factors such as chronic comorbidities. Individual-level socioeconomic indicators were also unavailable, though region partially addressed this limitation.

In conclusion, this study clarifies the long-term survival patterns of patients with pneumoconiosis by applying a competing risk model. Older age at diagnosis, silicosis, an earlier era of diagnosis, and advanced baseline stage were identified as independent risk factors for pneumoconiosis-related death. Compared with traditional survival analysis methods, the Fine–Gray model effectively mitigated the bias arising from competing events. These findings not only provide a novel methodological perspective for the prognostic evaluation of occupational diseases but also highlight the critical importance of implementing comprehensive health management strategies for patients with pneumoconiosis to control the risk of competing death.

## 5 Conclusion

This study identifies non-pneumoconiosis-related death as a critical competing risk in the long-term survival of patients with pneumoconiosis. The Fine–Gray model accounts for competing events and provides appropriate estimates for cumulative incidence prediction, addressing the limitations of traditional survival methods that may overestimate certain risk effects when competing risks are ignored. Independent predictors of pneumoconiosis-related death included age at diagnosis, regional economic level, disease type, era of diagnosis, and baseline stage. The observed mortality reduction in recent decades reflects the cumulative success of optimized occupational policies and clinical management. Given the rising dominance of non-pneumoconiosis deaths, particularly the systemic vulnerability seen in silicosis, future strategies must shift from singular disease prevention to comprehensive, full-life-cycle health management.

## Data Availability

The data analyzed in this study is subject to the following licenses/restrictions: the data can be obtained from the corresponding author upon reasonable request. Requests to access these datasets should be directed to hanlei_jscdc@163.com and liyeishere@jscdc.cn.
