# Supplementary material for: Long-term survival and the critical role of competing risks in pneumoconiosis: a large-scale retrospective cohort study
Source: Front Public Health. 2026 Mar 4;14:1782032. doi: 10.3389/fpubh.2026.1782032 (PMC12996100; doi:10.3389/fpubh.2026.1782032)
Supplement: Supplementary file 1 [file Table_1.docx]

Table S1. Baseline Characteristics of Included and Excluded Cases

| Variable | Included (n=18,064) | Excluded (n=9,175) | *P* value |
| --- | --- | --- | --- |
| Gender, n (%) |  |  | 0.9410 |
| Male | 16,875 (93.4) | 8,574 (93.4) |  |
| Female | 1,189 (6.6) | 601 (6.6) |  |
| Age at diagnosis, years, median (IQR) | 52.0 (45.0-62.0) | 55.0 (48.0-64.0) | <0.0001 |
| Industry, n (%) |  |  | <0.0001 |
| Mining | 9,000 (49.8) | 6,228 (67.9) |  |
| Manufacturing | 4,625 (25.6) | 2,230 (24.3) |  |
| Public/Social | 3,934 (21.8) | 415 (4.5) |  |
| Others | 505 (2.8) | 302 (3.3) |  |
| Dust exposure duration, years, median (IQR) | 15.0 (7.0-23.0) | 21.0 (14.0-28.0) | <0.0001 |
| Disease type, n (%) |  |  | <0.0001 |
| Silicosis | 12,275 (68.0) | 5,608 (61.1) |  |
| CWP | 3,100 (17.2) | 2,513 (27.4) |  |
| WP | 1,013 (5.6) | 105 (1.1) |  |
| Other pneumoconiosis | 1,676 (9.3) | 949 (10.3) |  |
| Stage at diagnosis, n (%) |  |  | <0.0001 |
| I | 15,338 (84.9) | 5,854 (63.8) |  |
| II | 2,158 (11.9) | 2,432 (26.5) |  |
| III | 568 (3.1) | 889 (9.7) |  |
| Era of diagnosis, n (%) |  |  | <0.0001 |
| Before 2000 | 5,883 (32.6) | 6,234 (67.9) |  |
| 2000-2010 | 6,475 (35.8) | 2,258 (24.6) |  |
| After 2010 | 5,706 (31.6) | 683 (7.4) |  |
| Region, n (%) |  |  | <0.0001 |
| Southern Jiangsu | 10,855 (60.1) | 5,634 (61.4) |  |
| Central Jiangsu | 665 (3.7) | 231 (2.5) |  |
| Northern Jiangsu | 6,544 (36.2) | 3,310 (36.1) |  |
| Missing | 0 (0.0) | 0 (0.0) |  |
| Follow-up time, years, median (IQR) | 17.0 (10.0-26.0) | 16.0 (8.0-25.0) | <0.0001 |
| Disease progression, n (%) |  |  | <0.0001 |
| No | 16,757 (92.8) | 7,941 (86.6) |  |
| Yes | 1,307 (7.2) | 1,234 (13.4) |  |

Abbreviations: IQR, interquartile range; CWP, coal workers' pneumoconiosis; WP, welder's pneumoconiosis. Note: Continuous variables are presented as median (IQR) and compared using the Mann-Whitney U test. Categorical variables are presented as n (%) and compared using Pearson's chi-square test. Region: Southern Jiangsu (Nanjing, Suzhou, Wuxi, Changzhou, Zhenjiang), Central Jiangsu (Nantong, Yangzhou, Taizhou), Northern Jiangsu (Xuzhou, Huai'an, Yancheng, Lianyungang, Suqian).
