# Supplementary material for: Long-term survival and the critical role of competing risks in pneumoconiosis: a large-scale retrospective cohort study
Source: Front Public Health. 2026 Mar 4;14:1782032. doi: 10.3389/fpubh.2026.1782032 (PMC12996100; doi:10.3389/fpubh.2026.1782032)
Supplement: Supplementary file 2 [file Table_2.docx]

Table S2: Follow-up Time by Era of Diagnosis and Stage at Diagnosis

A. Follow-up Time by Era of Diagnosis

| Era of Diagnosis | N | Median (years) | IQR (years) |
| --- | --- | --- | --- |
| Before 2000 | 5883 | 30.0 | 26.0-33.0 |
| 2000-2010 | 6475 | 17.0 | 15.0-21.0 |
| After 2010 | 5706 | 8.0 | 5.0-11.0 |

B. Follow-up Time by Stage at Diagnosis

| Stage at Diagnosis | N | Median (years) | IQR (years) |
| --- | --- | --- | --- |
| Stage I | 15338 | 17.0 | 11.0-27.0 |
| Stage II | 2158 | 17.0 | 10.0-23.0 |
| Stage III | 568 | 11.0 | 6.0-17.0 |

IQR, interquartile range. Follow-up time was calculated from the date of pneumoconiosis diagnosis to the date of death or last follow-up (December 31, 2024).
