# Supplementary material for: Long-term survival and the critical role of competing risks in pneumoconiosis: a large-scale retrospective cohort study
Source: Front Public Health. 2026 Mar 4;14:1782032. doi: 10.3389/fpubh.2026.1782032 (PMC12996100; doi:10.3389/fpubh.2026.1782032)
Supplement: Supplementary file 3 [file Table_3.docx]

Table S3. Fine-Gray competing risk regression analysis for competing death

| Variable | Unadjusted SHR (95% CI) | *P* value | Adjusted SHR (95% CI) | *P* value |
| --- | --- | --- | --- | --- |
| Age at diagnosis, years | 1.10 (1.09-1.10) | <0.001 | 1.09 (1.09-1.10) | <0.001 |
| Gender |  |  |  |  |
| Male | Reference |  | Reference |  |
| Female | 0.73 (0.61-0.89) | 0.001 | 0.77 (0.63-0.94) | 0.012 |
| Industry |  |  |  |  |
| Mining | Reference |  | Reference |  |
| Manufacturing | 0.69 (0.62-0.77) | <0.001 | 1.11 (0.96-1.28) | 0.140 |
| Public/Social | 2.98 (2.71-3.28) | <0.001 | 1.12 (0.95-1.32) | 0.170 |
| Others | 0.88 (0.66-1.17) | 0.380 | 1.24 (0.93-1.67) | 0.140 |
| Region |  |  |  |  |
| Southern Jiangsu | Reference |  | Reference |  |
| Central Jiangsu | 0.85 (0.66-1.10) | 0.220 | 1.44 (1.09-1.91) | 0.011 |
| Northern Jiangsu | 2.50 (2.29-2.73) | <0.001 | 1.90 (1.69-2.14) | <0.001 |
| Disease type |  |  |  |  |
| Silicosis | Reference |  | Reference |  |
| CWP | 0.56 (0.50-0.64) | <0.001 | 0.54 (0.46-0.64) | <0.001 |
| Welder's pneumoconiosis | 0.19 (0.10-0.34) | <0.001 | 0.49 (0.27-0.90) | 0.020 |
| Other pneumoconiosis | 0.91 (0.79-1.06) | 0.230 | 1.07 (0.90-1.28) | 0.450 |
| Era of diagnosis |  |  |  |  |
| Before 2000 | Reference |  | Reference |  |
| 2000-2010 | 1.51 (1.37-1.66) | <0.001 | 0.98 (0.87-1.11) | 0.770 |
| After 2010 | 3.10 (2.75-3.48) | <0.001 | 0.93 (0.78-1.10) | 0.400 |
| Stage at diagnosis |  |  |  |  |
| I | Reference |  | Reference |  |
| II | 1.39 (1.23-1.57) | <0.001 | 1.49 (1.29-1.72) | <0.001 |
| III | 1.85 (1.48-2.30) | <0.001 | 1.41 (1.10-1.80) | 0.006 |
| Dust exposure duration, years | 0.98 (0.97-0.98) | <0.001 | 0.99 (0.98-1.00) | <0.001 |

Abbreviations: IQR, interquartile range; CWP, coal workers' pneumoconiosis; WP, welder's pneumoconiosis. Notes: Pneumoconiosis-related death is the event of interest. Non-pneumoconiosis-related death is the competing event. P values were calculated using the Kruskal-Wallis test for continuous variables and Pearson's chi-square test for categorical variables.
