# Supplementary material for: Long-term survival and the critical role of competing risks in pneumoconiosis: a large-scale retrospective cohort study
Source: Front Public Health. 2026 Mar 4;14:1782032. doi: 10.3389/fpubh.2026.1782032 (PMC12996100; doi:10.3389/fpubh.2026.1782032)
Supplement: Supplementary file 4 [file Table_4.docx]

Table S4. Comparison of Cox and Fine-Gray regression models for pneumoconiosis-related death

| Variable | Cox HR (95% CI) | *P* value | Fine-Gray SHR (95% CI) | *P* value | Relative Bias (%) |
| --- | --- | --- | --- | --- | --- |
| Age at diagnosis, years | 1.12 (1.11-1.14) | <0.001 | 1.09 (1.08-1.1) | <0.001 | +3.5 |
| Gender |  |  |  |  |  |
| Male | Reference |  | Reference |  |  |
| Female | 0.86 (0.63-1.18) | 0.358 | 0.96 (0.69-1.32) | 0.780 | -9.6 |
| Industry |  |  |  |  |  |
| Mining | Reference |  | Reference |  |  |
| Manufacturing | 0.94 (0.74-1.2) | 0.616 | 0.92 (0.71-1.19) | 0.530 | +2.1 |
| Public/Social | 0.67 (0.5-0.9) | 0.009 | 0.66 (0.47-0.92) | 0.014 | +1.9 |
| Others | 1.01 (0.57-1.81) | 0.967 | 0.86 (0.48-1.56) | 0.630 | +17.1 |
| Disease type |  |  |  |  |  |
| Silicosis | Reference |  | Reference |  |  |
| CWP | 0.52 (0.4-0.68) | <0.001 | 0.62 (0.49-0.79) | <0.001 | -15.8 |
| Welder's pneumoconiosis | 0.99 (0.4-2.44) | 0.981 | 0.82 (0.33-2.04) | 0.680 | +19.9 |
| Other pneumoconiosis | 1.07 (0.79-1.46) | 0.657 | 1.00 (0.72-1.4) | 0.980 | +6.9 |
| Era of diagnosis |  |  |  |  |  |
| Before 2000 | Reference |  | Reference |  |  |
| 2000-2010 | 0.89 (0.71-1.12) | 0.328 | 0.66 (0.54-0.8) | <0.001 | +36.0 |
| After 2010 | 0.62 (0.45-0.87) | 0.005 | 0.38 (0.28-0.52) | <0.001 | +65.6 |
| Stage at diagnosis |  |  |  |  |  |
| I | Reference |  | Reference |  |  |
| II | 3.31 (2.74-4) | <0.001 | 2.94 (2.41-3.6) | <0.001 | +12.5 |
| III | 5.45 (4.2-7.08) | <0.001 | 5.18 (3.91-6.87) | <0.001 | +5.2 |
| Region |  |  |  |  |  |
| Southern Jiangsu | Reference |  | Reference |  |  |
| Central Jiangsu | 1.96 (1.27-3) | 0.002 | 1.92 (1.24-2.98) | 0.004 | +1.9 |
| Northern Jiangsu | 1.40 (1.14-1.73) | 0.001 | 1.44 (1.16-1.78) | <0.001 | -2.3 |
| Dust exposure duration, years | 1.00 (0.99-1.01) | 0.466 | 1.01 (1-1.02) | 0.160 | -0.4 |

Abbreviations: HR, hazard ratio; SHR, subdistribution hazard ratio; CI, confidence interval; CWP, coal workers' pneumoconiosis. Cox model treats competing events (non-pneumoconiosis deaths) as censored. Fine-Gray model accounts for competing risks using subdistribution hazard approach. Relative bias = (Cox HR - Fine-Gray SHR) / Fine-Gray SHR × 100%. Positive bias indicates Cox model overestimates the effect; negative bias indicates underestimation.
