# Supplementary figures and images for: Long-term survival and the critical role of competing risks in pneumoconiosis: a large-scale retrospective cohort study
Source: Front Public Health. 2026 Mar 4;14:1782032. doi: 10.3389/fpubh.2026.1782032 (PMC12996100; doi:10.3389/fpubh.2026.1782032)

**A**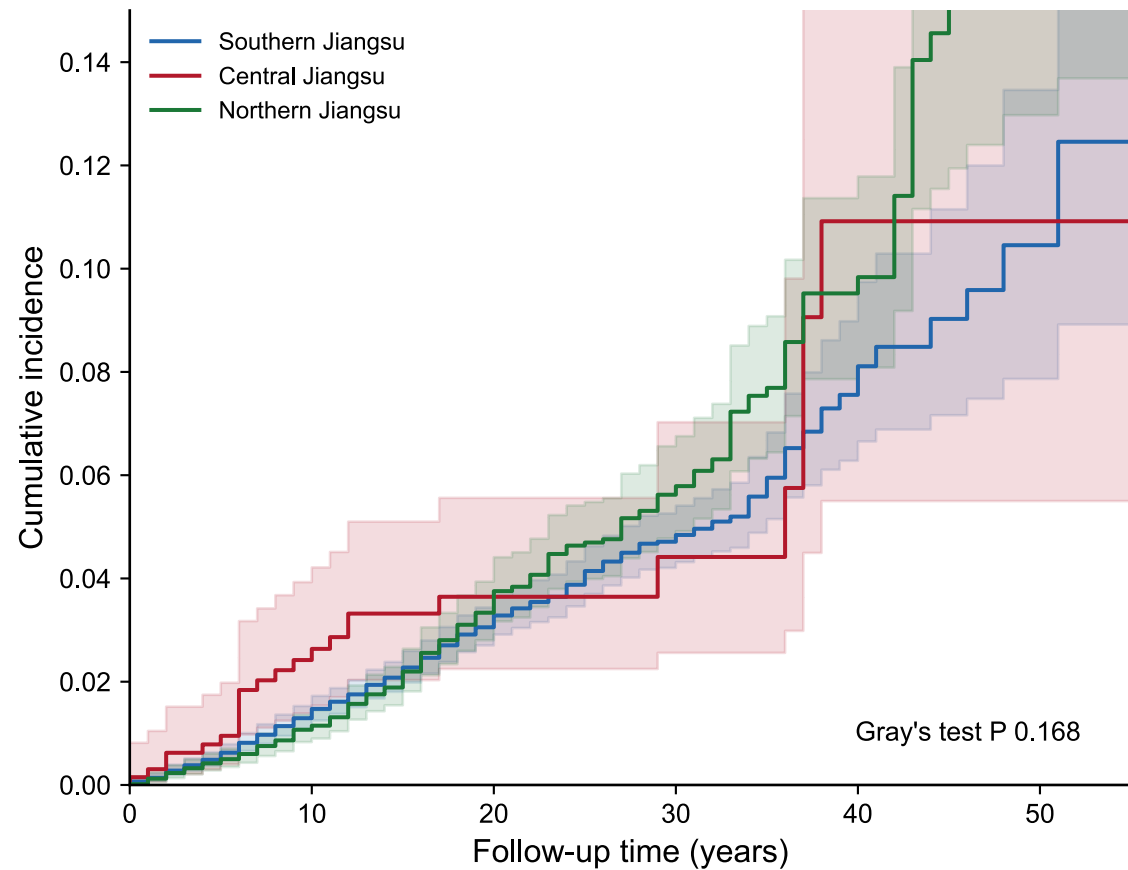**B**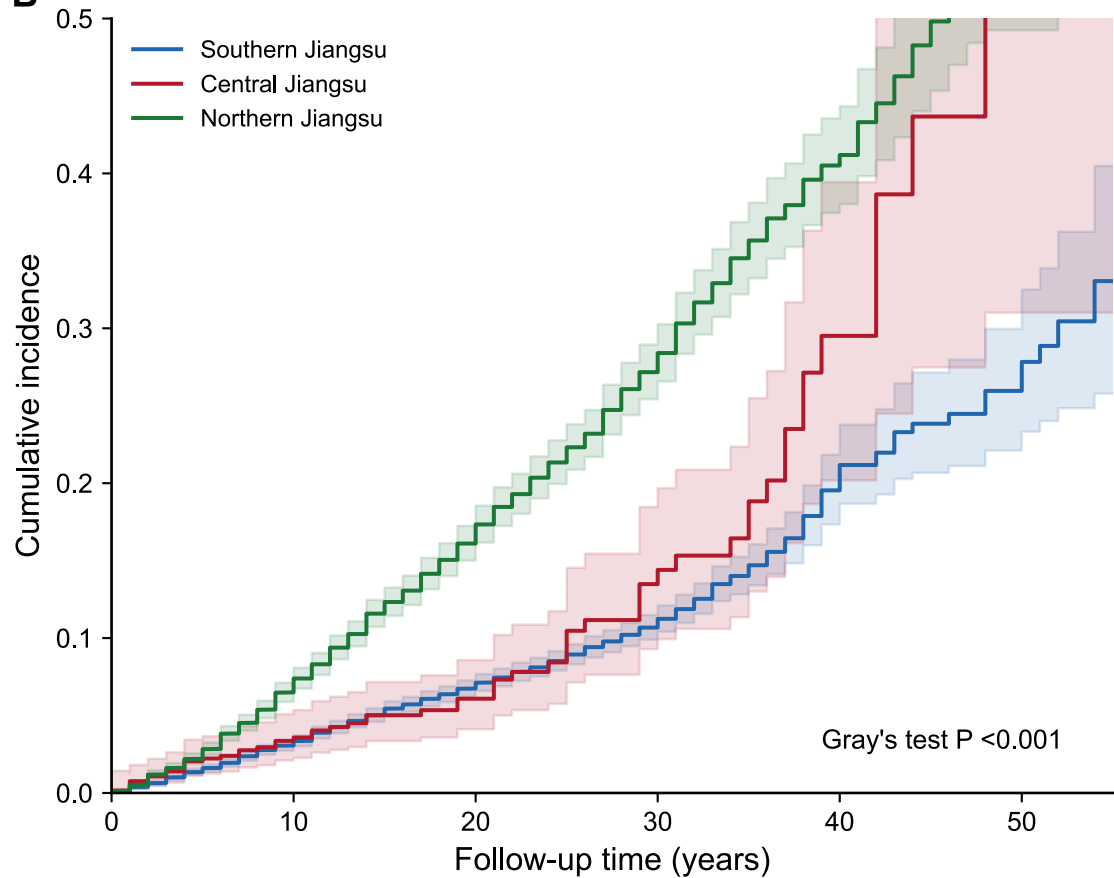

Supplement: Supplementary file 5 [file Data_Sheet_1.pdf]

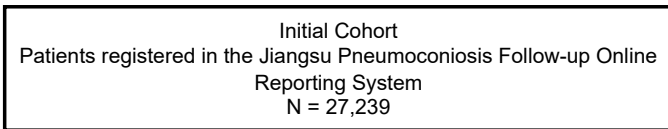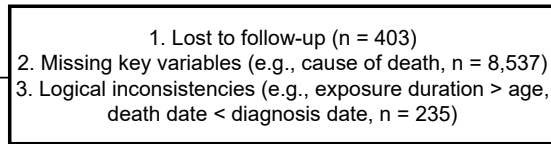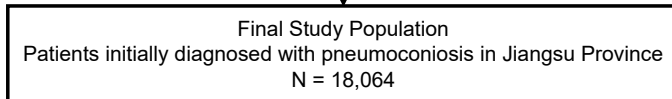

Supplement: Supplementary file 6 [file Data_Sheet_2.pdf]

**A**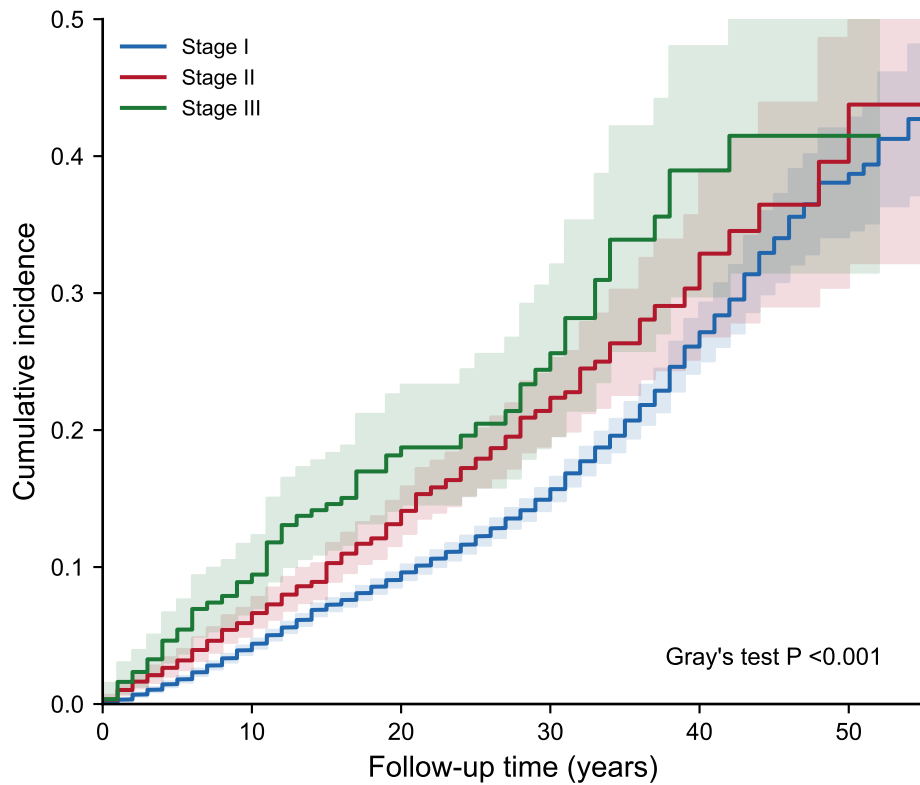**B**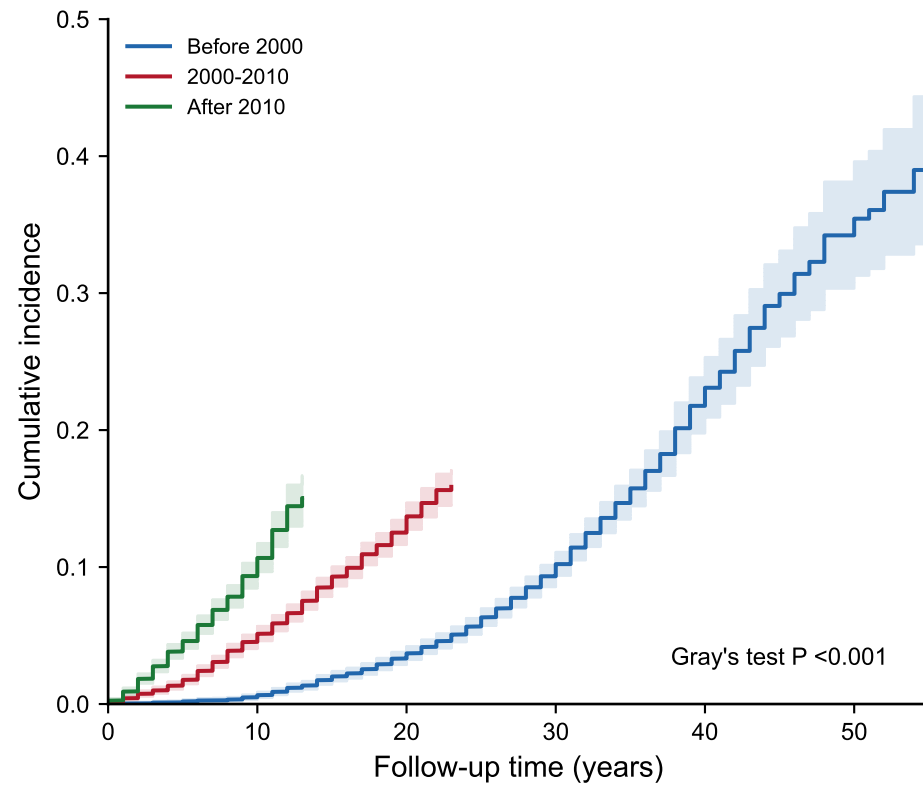**C**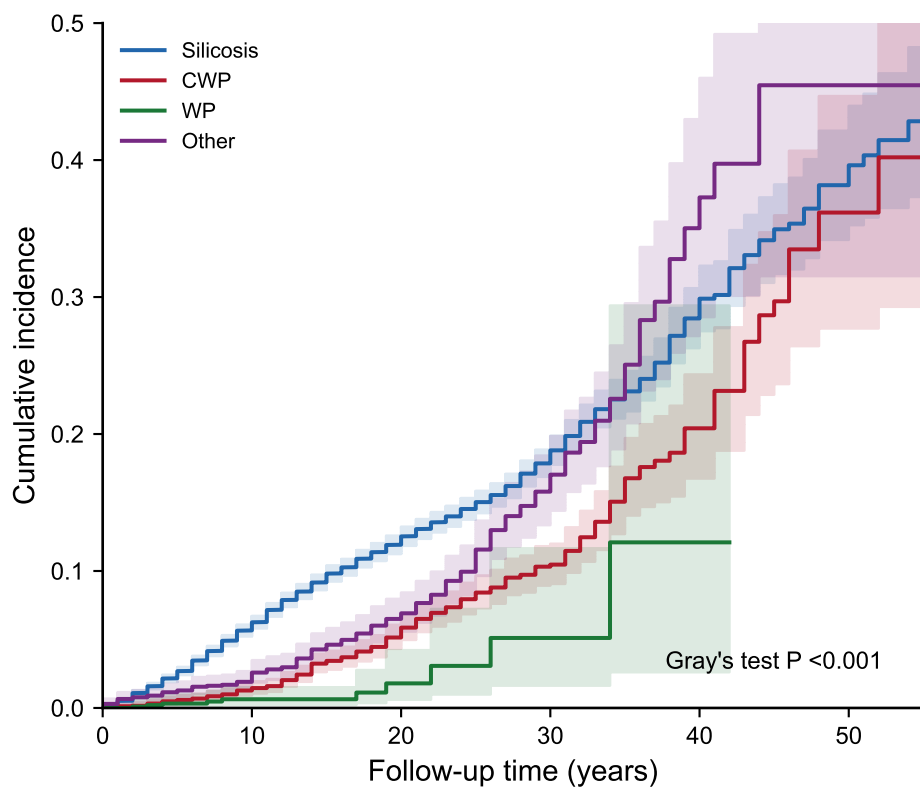**D**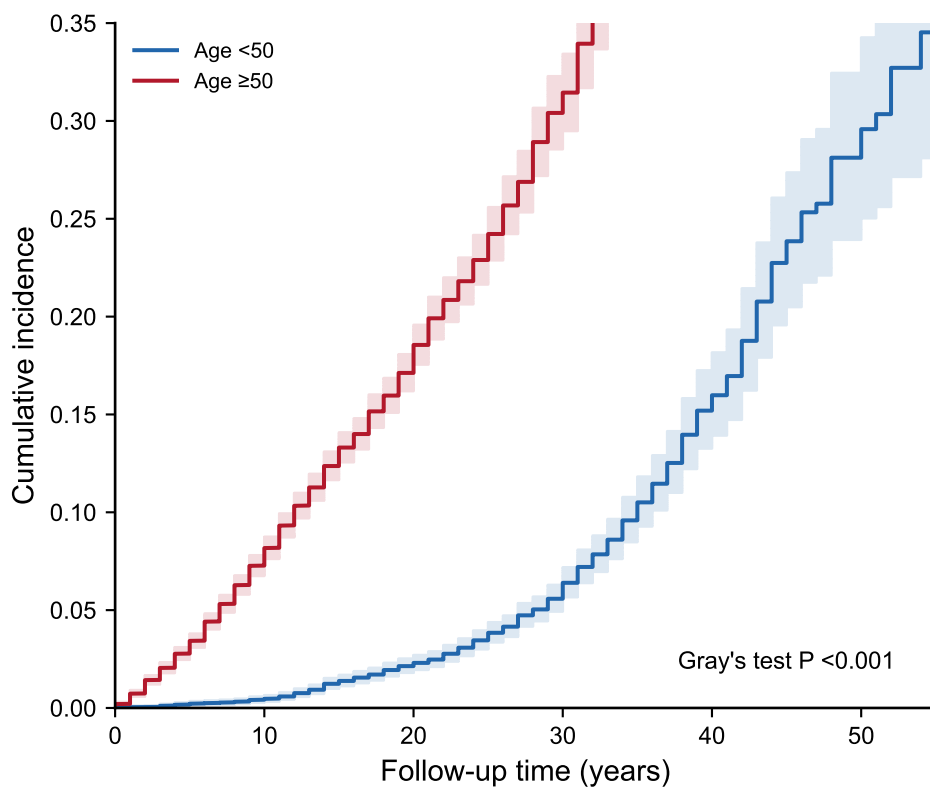

Supplement: Supplementary file 7 [file Data_Sheet_3.pdf]

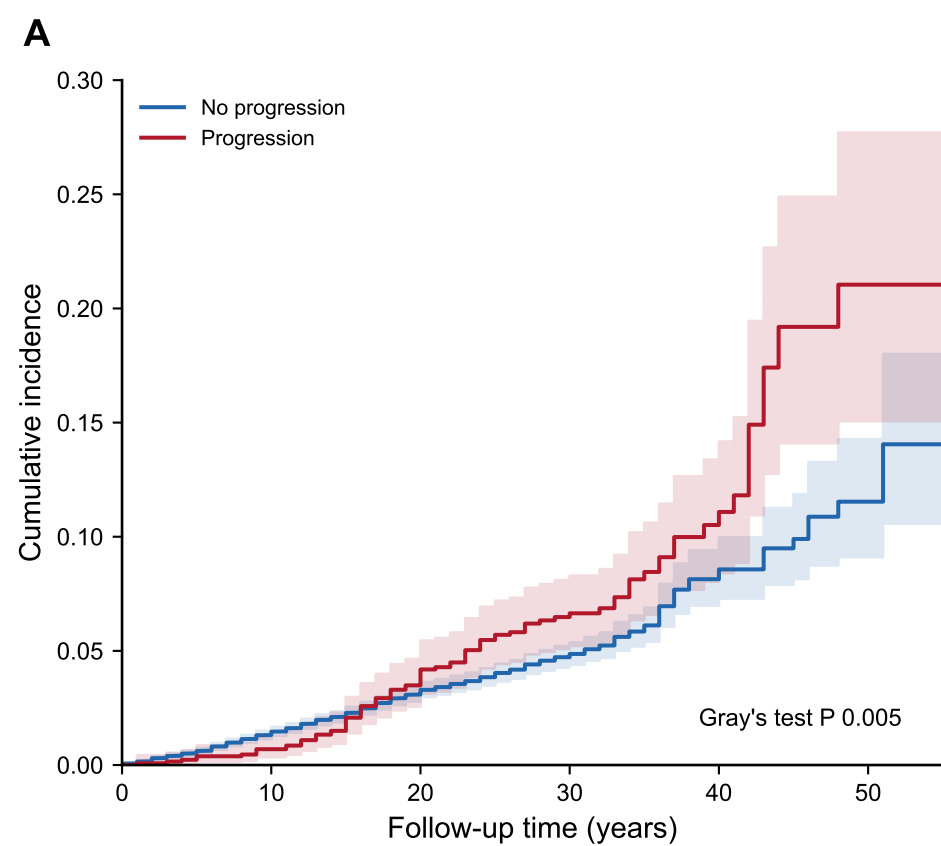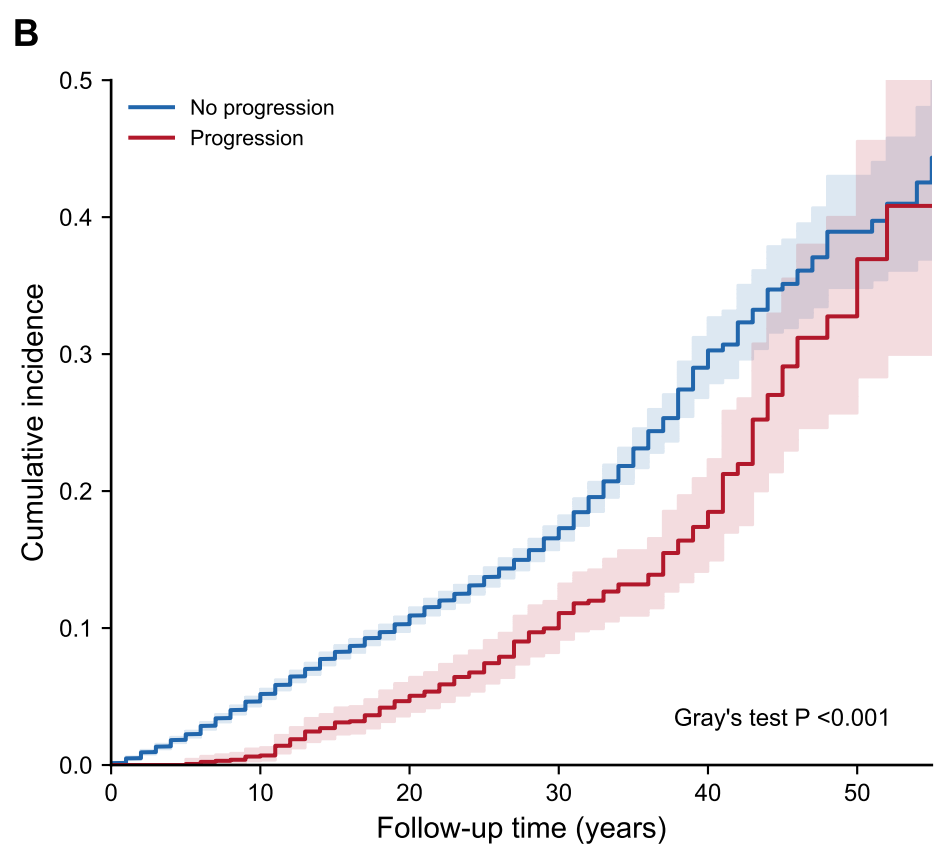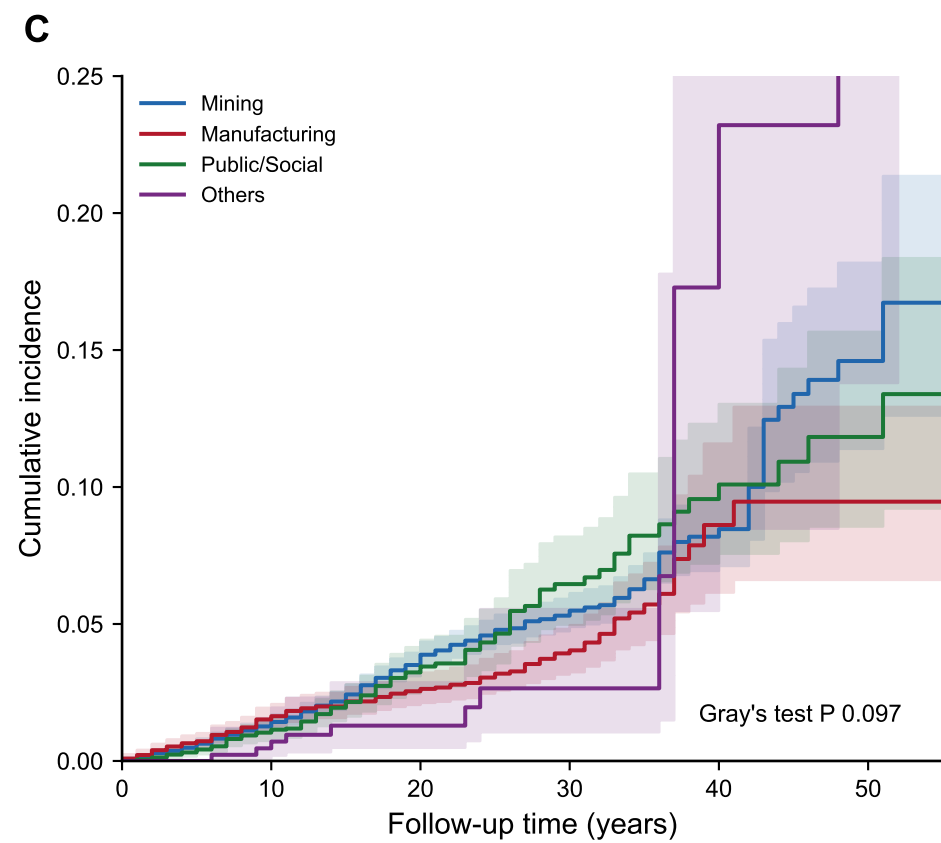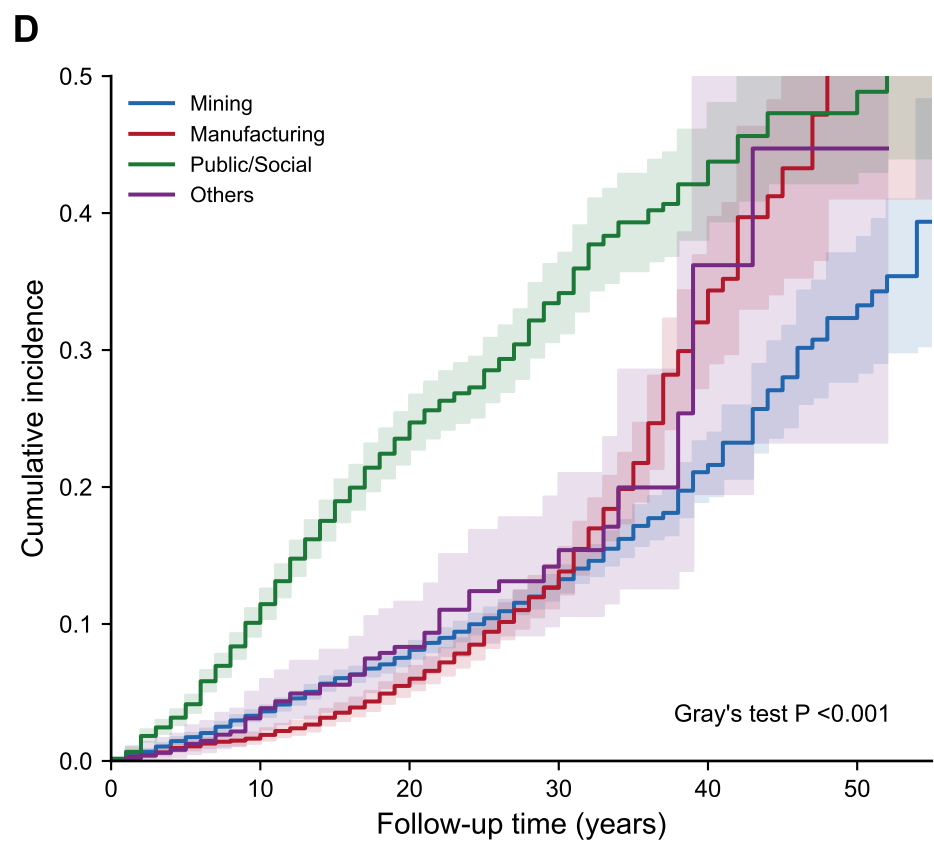

Supplement: Supplementary file 8 [file Data_Sheet_4.pdf]

A

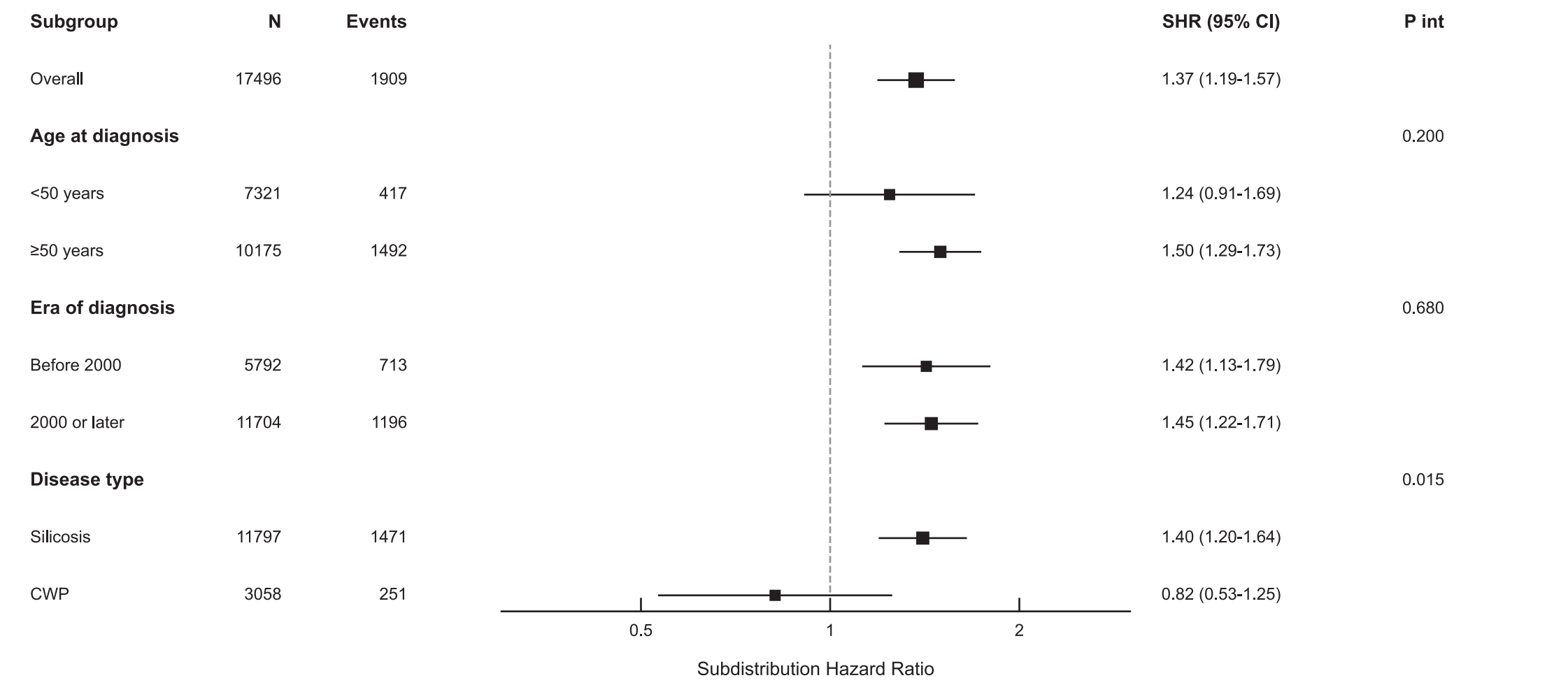

B

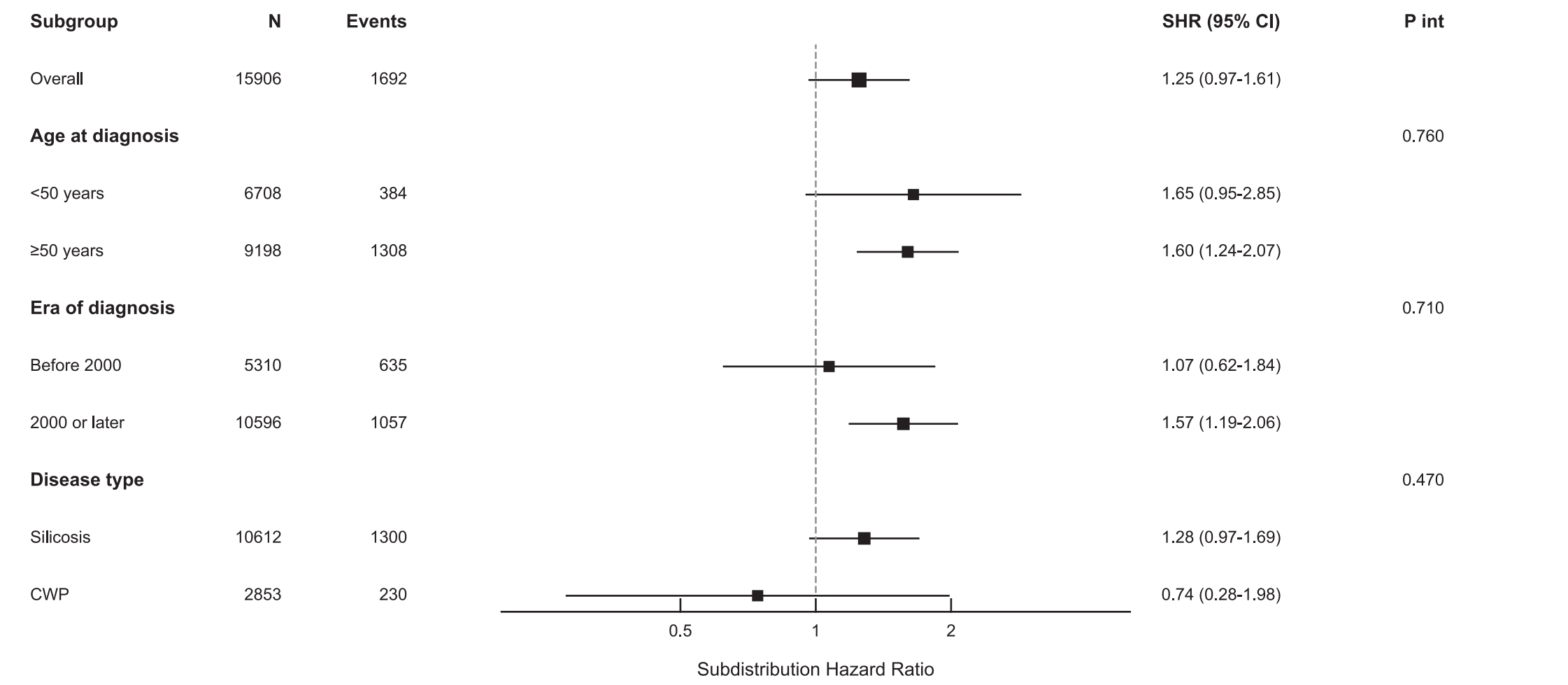

Supplement: Supplementary file 9 [file Data_Sheet_5.pdf]

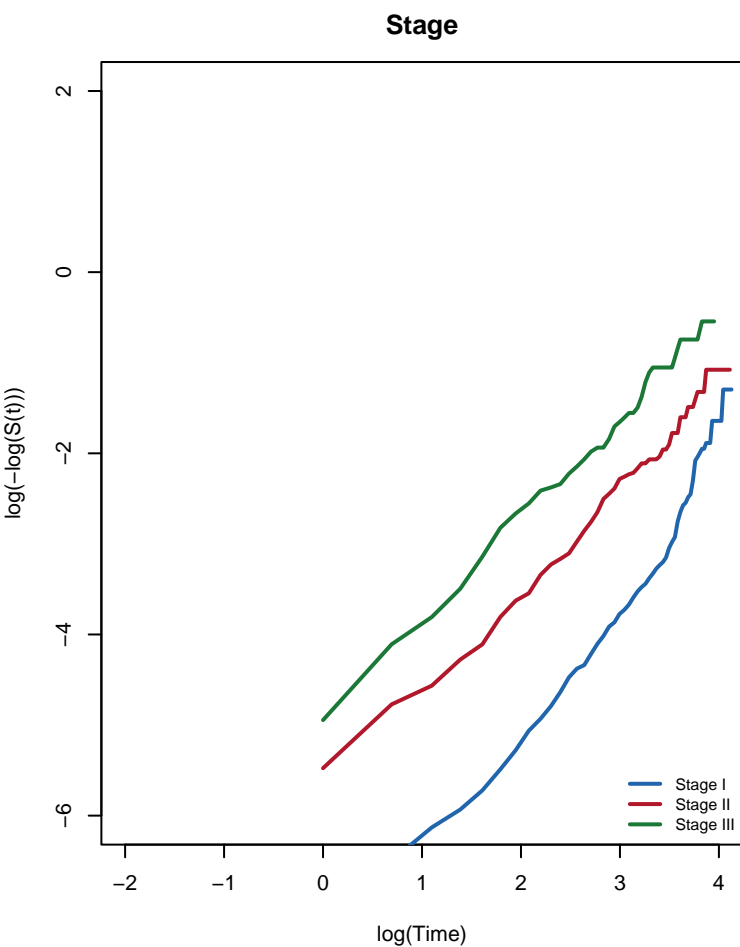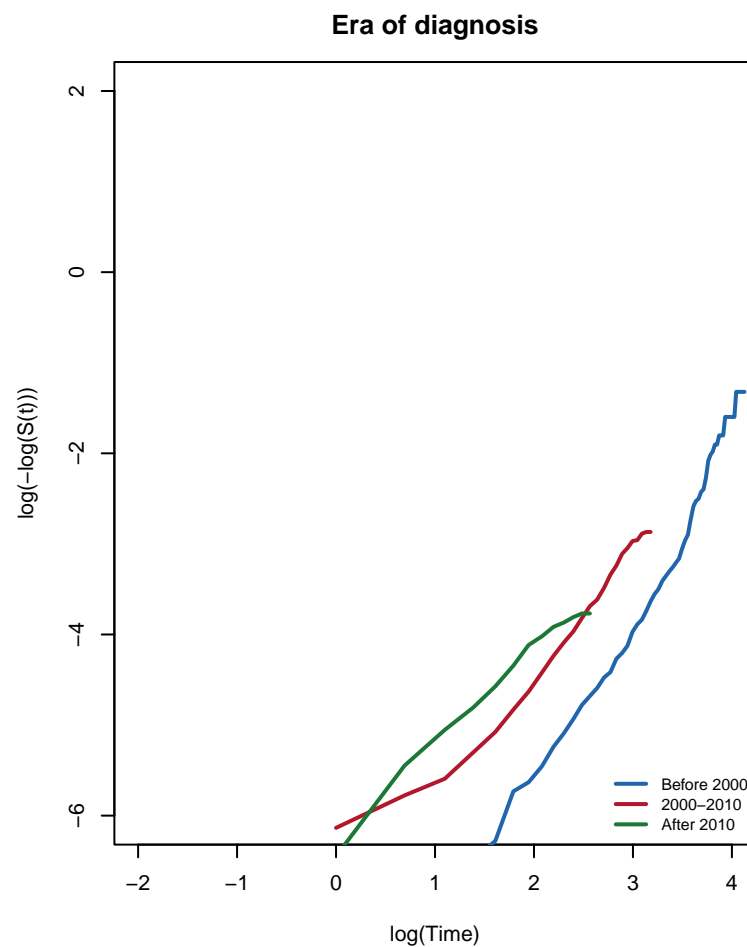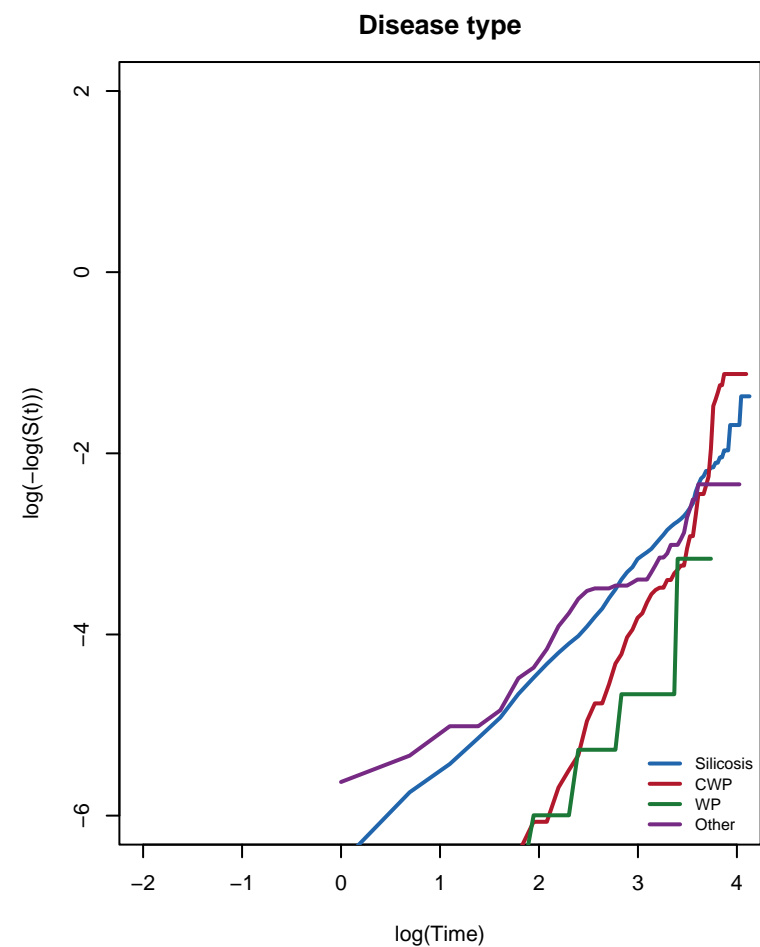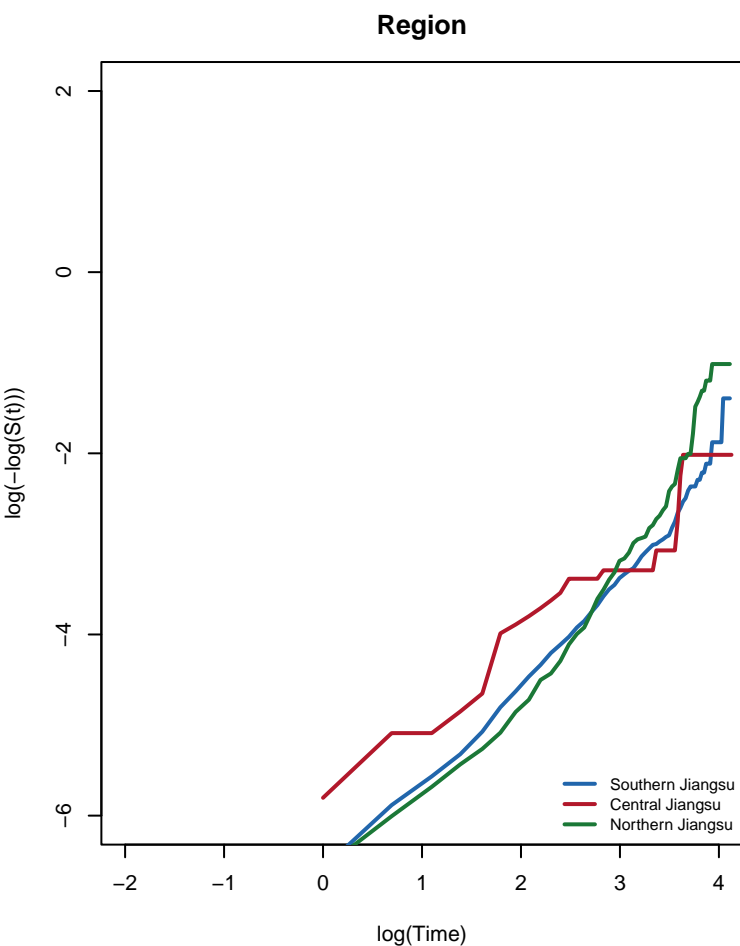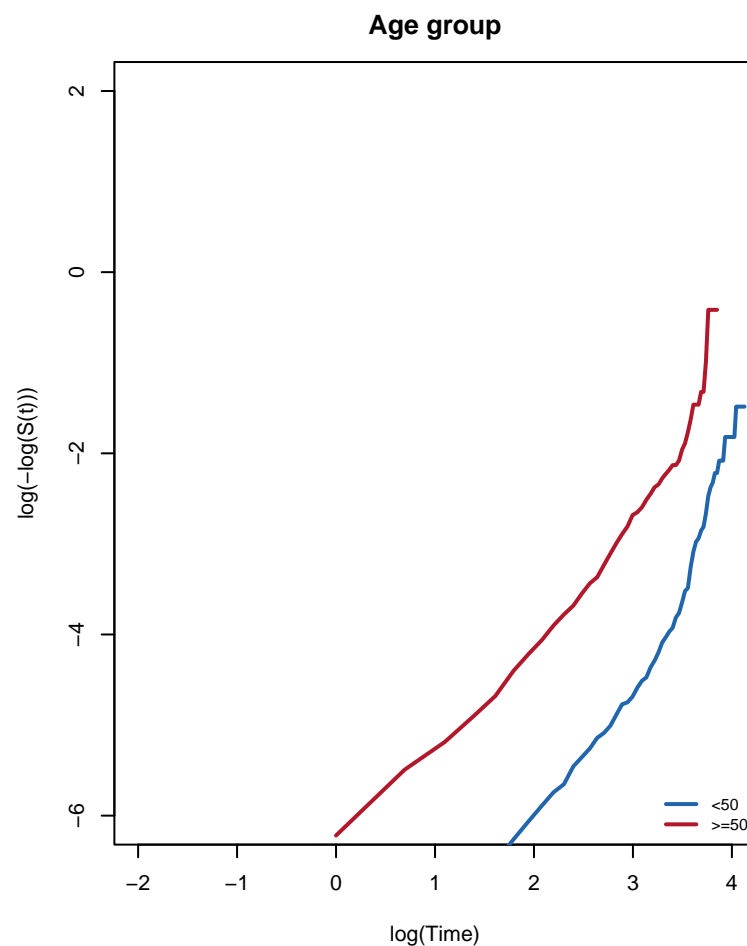

Supplement: Supplementary file 10 [file Data_Sheet_6.pdf]

**3-Year Calibration**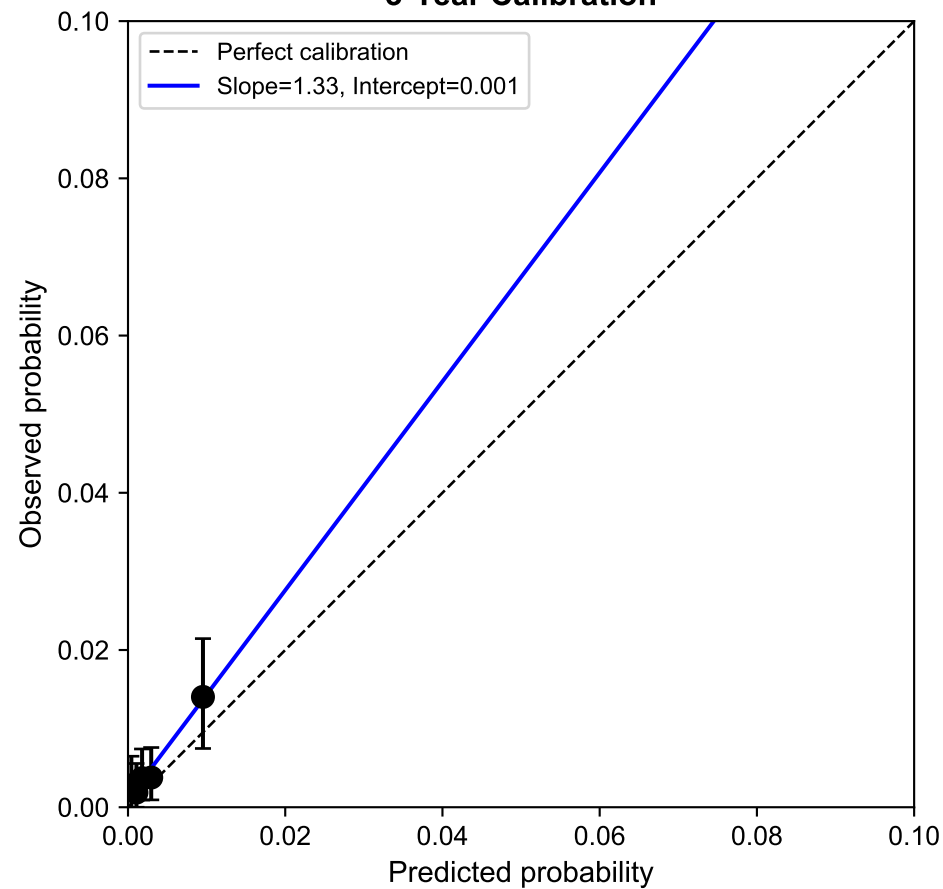**5-Year Calibration**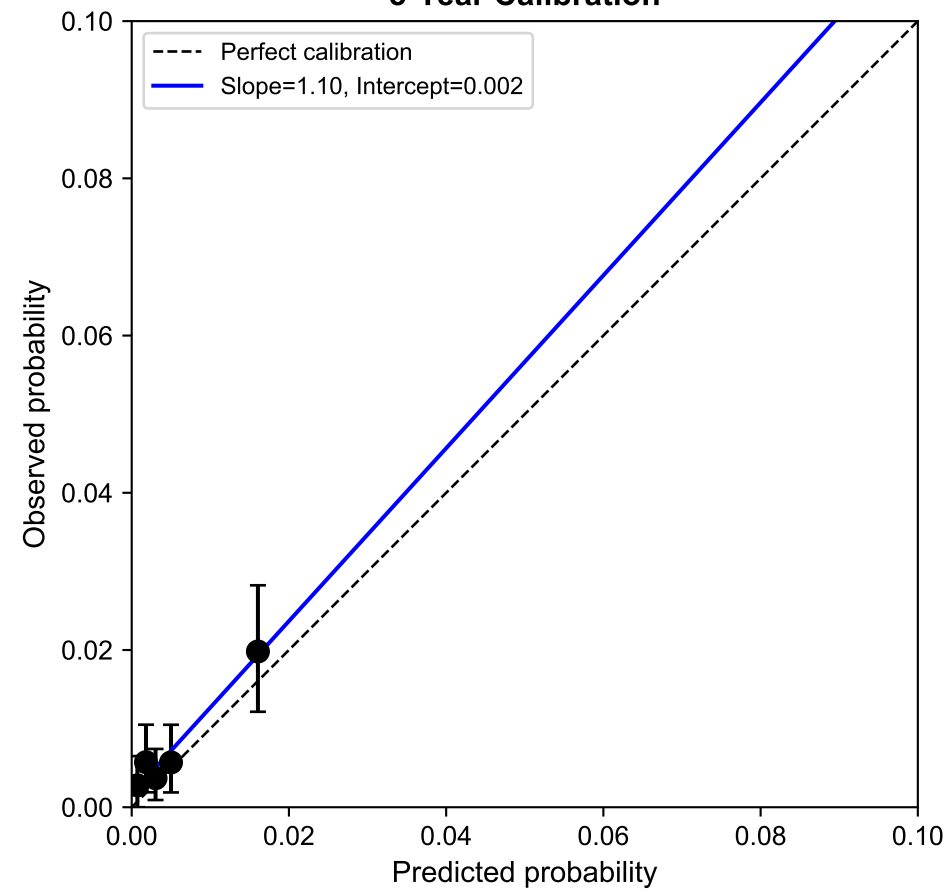**10-Year Calibration**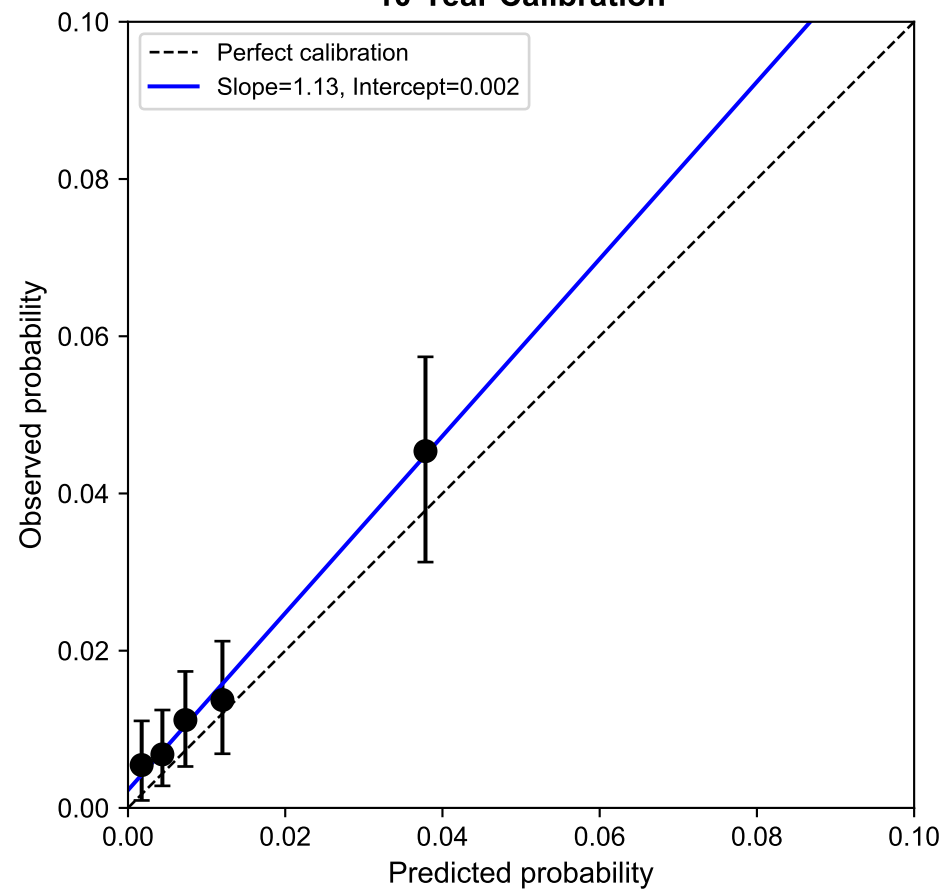**15-Year Calibration**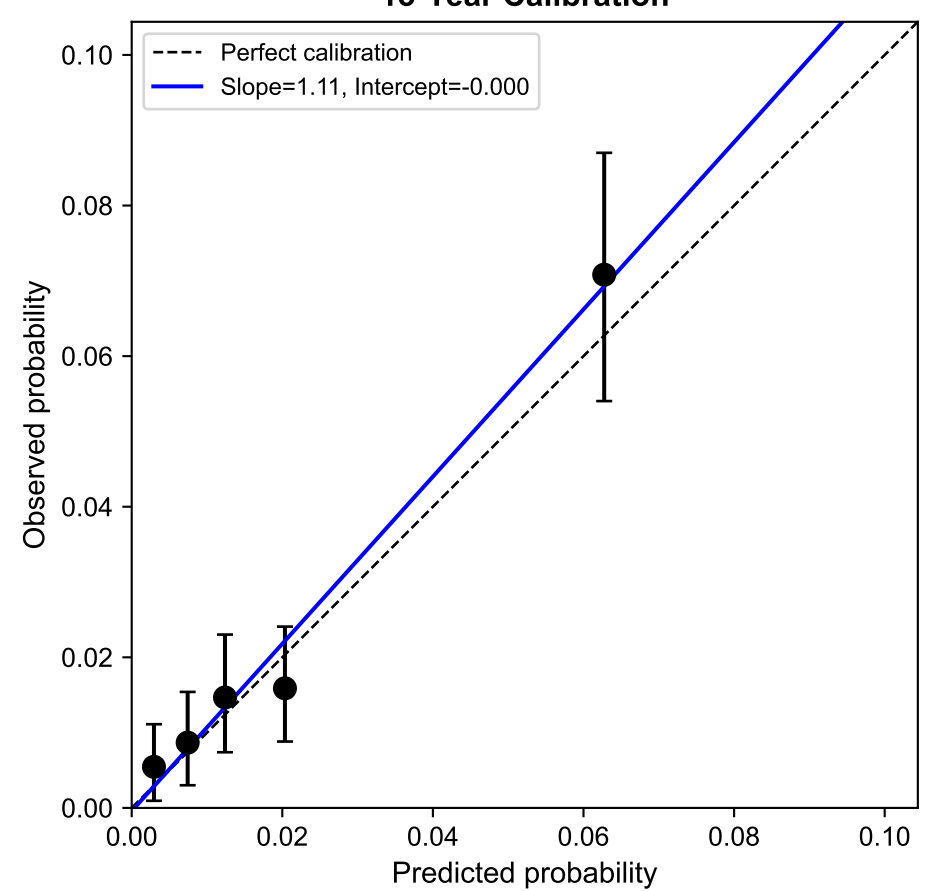

Supplement: Supplementary file 12 [file Data_Sheet_8.pdf]
