# Supplementary material for: Long-term survival and the critical role of competing risks in pneumoconiosis: a large-scale retrospective cohort study
Source: Front Public Health. 2026 Mar 4;14:1782032. doi: 10.3389/fpubh.2026.1782032 (PMC12996100; doi:10.3389/fpubh.2026.1782032)
Supplement: Supplementary file 11 [file Data_Sheet_7.pdf]

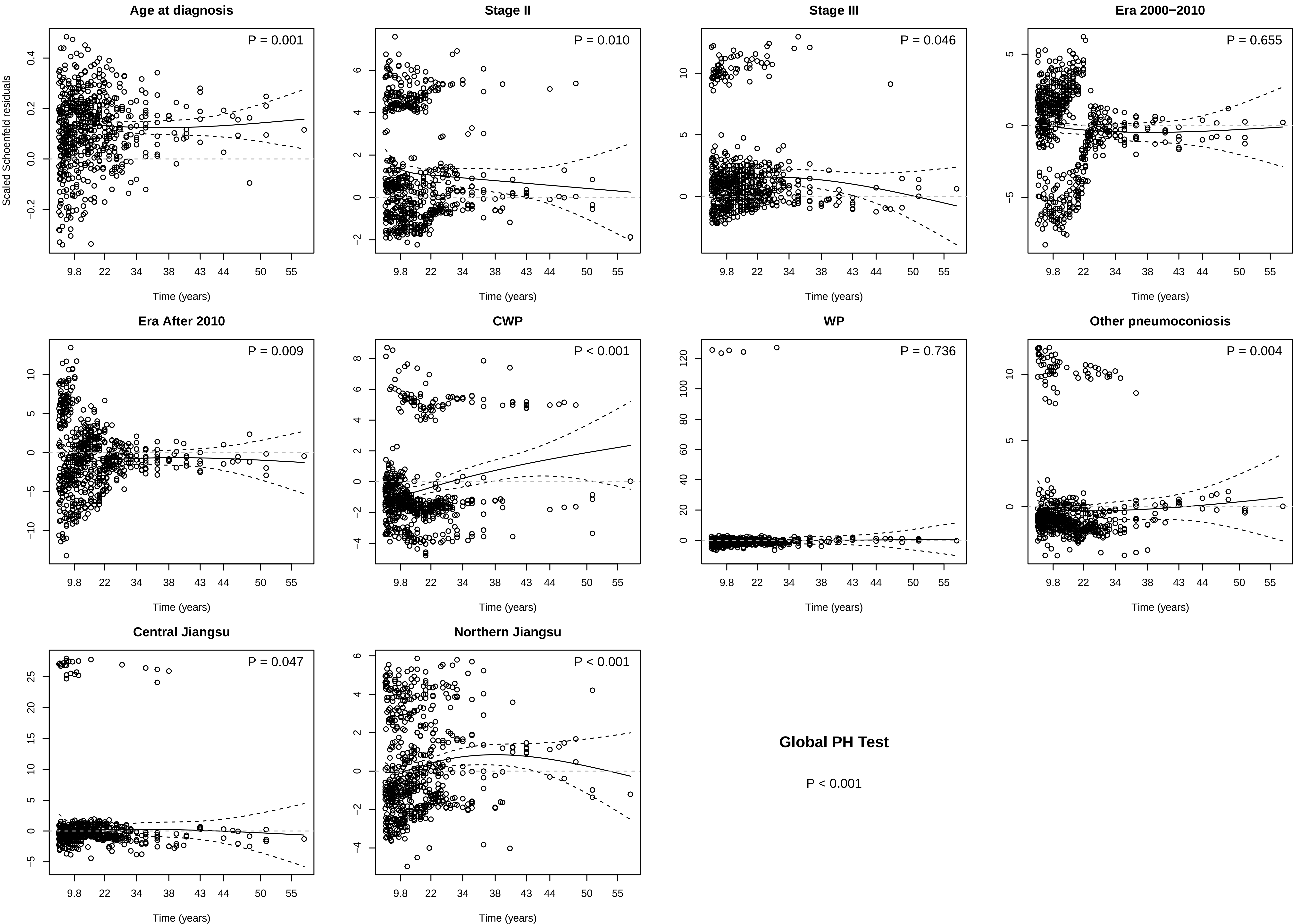
Age at diagnosis
P = 0.001
Stage II
P = 0.010
Stage III
P = 0.046
Era 2000−2010
P = 0.655
Era After 2010
P = 0.009
CWP
P < 0.001
WP
P = 0.736
Other pneumoconiosis
P = 0.004
Central Jiangsu
P = 0.047
Northern Jiangsu
P < 0.001
Scaled Schoenfeld residuals
Time (years)
Global PH Test
P < 0.001
